# Neural defensive circuits underlie helping under threat in humans

**DOI:** 10.7554/eLife.78162

**Published:** 2022-10-25

**Authors:** Joana B Vieira, Andreas Olsson

**Affiliations:** 1 Department of Psychology, Faculty of Health and Life Sciences, University of Exeter Exeter United Kingdom; 2 Department of Clinical Neuroscience, Karolinska Institutet Stockholm Sweden; University of Maryland United States; University College London United Kingdom

**Keywords:** Fear, altruism, Brain, Human

## Abstract

Empathy for others’ distress has long been considered the driving force of helping. However, when deciding to help others in danger, one must consider not only their distress, but also the risk to oneself. Whereas the role of self-defense in helping has been overlooked in human research, studies in other animals indicate defensive responses are necessary for the protection of conspecifics. In this pre-registered study (N=49), we demonstrate that human defensive neural circuits are implicated in helping others under threat. Participants underwent fMRI scanning while deciding whether to help another participant avoid aversive electrical shocks, at the risk of also being shocked. We found that higher engagement of neural circuits that coordinate fast escape from self-directed danger (including the insula, PAG, and ACC) facilitated decisions to help others. Importantly, using representational similarity analysis, we found that the strength with which the amygdala and insula uniquely represented the threat to oneself (and not the other’s distress) predicted helping. Our findings indicate that in humans, as other mammals, defensive mechanisms play a greater role in helping behavior than previously understood.

## Introduction

Helping someone in danger (e.g., by saving a person who fell on the train tracks, or running into a building in flames to rescue someone inside) may expose oneself to health and life-threatening risks. Nevertheless, such helping behaviors are observed across species (Hernandez-Lallement et al., 2020; Oliner, 2004; Preston, 2013; Rand and Epstein, 2014; de Waal and Lanting, 1997). Risky helping differs from other altruistic actions in that it occurs in the simultaneous presence of two highly salient cues: the distress of a conspecific in need, and a potential threat to the self. In humans, a wealth of research has been dedicated to the former, explaining how perceiving distress in others may trigger the motivation to help (Batson et al., 1987; de Waal and Preston, 2017), particularly if the helper is not under threat themselves (Preston, 2013). But virtually nothing is known about how, in a threatening situation, one’s own responses to the threat may drive decisions to help. More so, animal research suggests defensive brain mechanisms may in fact be implicated in aiding or protecting conspecifics (Ben-Ami Bartal et al., 2016; Bosch, 2013; Rickenbacher et al., 2017). Understanding the neurocognitive processes underlying the motivation to both safeguard oneself and helping others is critical to explain inter-individual behavior in dangerous contexts. The overarching goal of our study was thus to determine how one’s own defensive responses to threat guide decisions to help others in dangerous situations.

In humans, defensive responses to threat are graded as a function of the proximity or imminence of the threatening stimulus, paralleling predatory avoidance responses in other mammals (Fanselow and Lester, 1988; Mobbs et al., 2020). Distal and unpredictable threats are typically associated with risk assessment and intermittent anxiety, allowing for slower and more flexible escape decisions. As threat imminence increases and an attack becomes more likely, fixed and species-specific responses are triggered, such as freezing or, if immediate avoidance is necessary, fight-or-flight. Some behavioral reports indicate that different states along the defensive continuum may have dissociable effects on prosocial behavior. For example, following acute social stress, participants behave more prosocially in economic games (Tomova et al., 2017; von Dawans et al., 2012; von Dawans et al., 2019), make more moral decisions (Singer et al., 2017), and show greater empathy for others (Tomova et al., 2017). Importantly, it has been shown that individuals were more likely to help a co-participant avoid aversive electrical shocks when the threat of shock was imminent rather than distal (Vieira et al., 2020). This behavioral pattern was accompanied by faster reaction times and heart rate during imminent compared to distal threats, paralleling what has been found in response to imminent self-directed threats (Hashemi et al., 2019; Roelofs, 2017). Consistent with these laboratorial studies, higher danger in real-life situations (captured via public surveillance footage) has been associated with higher likelihood of bystander intervention (Lindegaard et al., 2022). Taken together, these findings suggest that defensive states triggered by high threat imminence may not only enable fast avoidance of self-directed threats, but also motivate helping when others are under threat. Yet, the neural basis of these effects is unclear. Specifically, it is unknown how the activation of specific sub-circuits underlying different defensive states (e.g., freezing vs. fight-or-flight) impacts decisions toward others in a threatening context.

**Figure 1.**
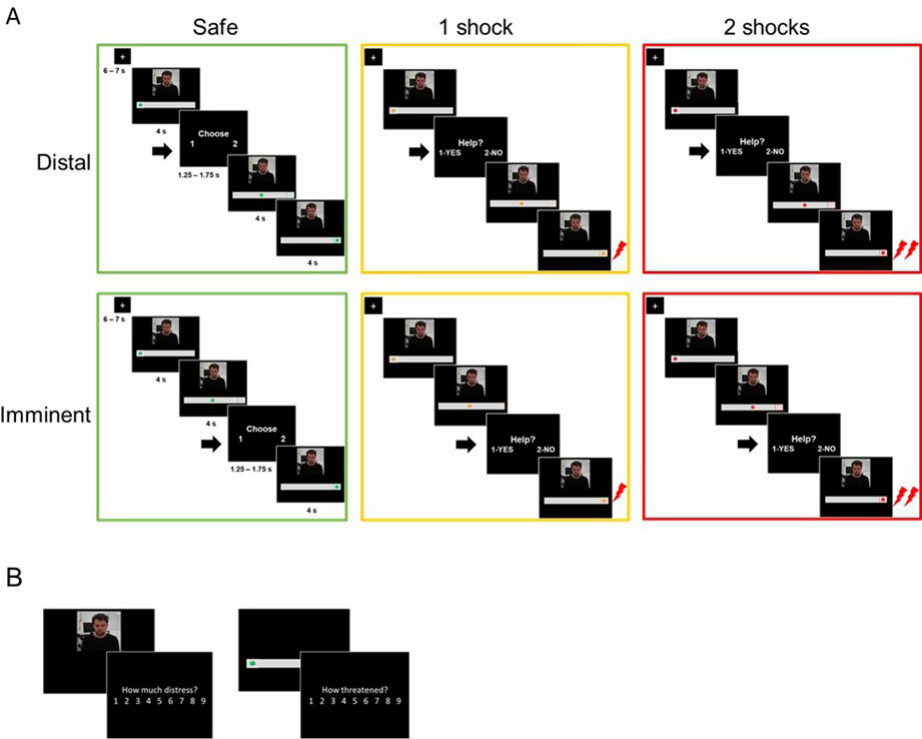
Outline of the experimental tasks. (**A**) fMRI helping under threat task. Participants saw the co-participant on the screen, together with a visual cue signaling threat (an upcoming shock). There were three threat levels: safe (0 shocks, green circle), moderate threat (1 shock, yellow circle), and high threat (2 shocks, red circle). In each trial of the task, the circle started static on the left (4 s), and then moved to the right (4 s). Participants were prompted to decide whether they wanted to help the co-participant or not (1.25–1.75 s) either in the beginning of trial (distal) or right before the moment of shock delivery (imminent). Therefore, the available time to make a decision was identical in distal and imminent threats. If participants decided to help, there was a 70% chance both themselves and co-participant would receive shocks; if they decided not to help, the co-participant would always receive a shock, and the participant would not. Decisions prompted on safe trials were to arbitrarily choose to press 1 or 2, since no shocks would be administered. (**B**) After the fMRI task, outside the scanner, participants re-watched clips of the co-participant presented during the scan, and were asked to rate how much ‘discomfort, anxiety or uneasiness’ he was experiencing in each clip on a 9-point scale. They also presented images of the threat cues and asked to rate, on the same scale, how threatened they felt themselves when they saw those images during the scan.

We aimed to characterize the involvement of different defensive neural responses on helping under threat. To do so, we used a paradigm adapted from Vieira et al., 2020, in which participants make helping decisions at different stages of threat imminence (details in Figure 1A). Briefly, a participant is asked to decide whether or not to help a co-participant (in reality, a confederate) avoid aversive electrical shocks. In each trial, the participant watches a supposedly live video-feed of the co-participant, and a visual cue signaling an upcoming shock. The participant is asked to decide whether to help the co-participant avoid the shock at the risk of receiving a shock from her/himself. These decisions are prompted in some trials in the beginning of the trial (*distal threat*), and in others immediately prior to the shock delivery (*imminent threat*). If the participant decides not to help, the co-participant always receives a shock; if the participant decides to help, there is a fixed probability both participant and co-participant will receive a shock.

Our hypotheses were guided by previous work on neural responses to the imminence of self-directed threats. It has been shown that the response to distal threats (i.e., unpredictable, spatially distant, retreating, slow moving) is coordinated by so-called ‘cognitive fear’ circuits, which include the ventromedial prefrontal cortex (vmPFC) and hippocampus (Mobbs et al., 2020; Qi et al., 2018). Conversely, imminent threats (i.e., predictable, spatially close, looming, and fast moving) predominantly engage ‘reactive’ fear circuits, which include the dorsal anterior cingulate (dACC), insula, and periaqueductal gray (PAG) (Mobbs et al., 2020; Qi et al., 2018). The amygdala plays a central role in both circuits, namely by coordinating adaptive switches between defensive states as a function of threat imminence (e.g., from freezing to fight-or-flight), through oxytocin-mediated communication between its central (CeA) and basolateral nucleus (BLA) (Terburg et al., 2018; Tovote et al., 2016). Based on these findings, we expected neural activation within the full defensive circuitry to respond to the threat level of the trial (i.e., safe, 1 shock, and 2 shocks), with higher engagement of brain regions previously included in cognitive fear circuits (i.e., vmPFC and hippocampus) in response to distal threats, and higher engagement of regions included in reactive fear circuits (i.e., insula, dACC, and PAG) in response to imminent threats. At the behavioral level, prior findings (Vieira et al., 2020) showed that helping decisions were more frequent under imminent than distal trials, suggesting that the activation of reactive fear circuits would facilitate helping behavior. We thus predicted that helping decisions would be associated with higher engagement of brain regions included in reactive fear circuits (i.e., amygdala, insula, ACC, and PAG).

One important aspect of our paradigm was that, as in most real-life dangerous situations, the threat and the conspecific in need were simultaneously presented. To dissociate the role of representations of threat and of other’s distress on helping behavior, after the scan we asked participants to rate the degree of distress experienced by *the co-participant* in each clip showed during the scan; also, participants rated how threatened they felt *themselves* when they saw the visual threat cues during the scan. These ratings were used as behavioral models in a representational similarity analysis (RSA; see Materials and methods) that identified neural representations of other’s distress and of threat to the self, and determined their association with helping behavior. It should be noted that our goal was specifically related to the link between neural representations of distress and threat, and helping behavior, and not to the dissociation of representations of distress and threat in the brain. This would be a highly interesting question to examine in future research.

The demonstration that neural representations of other’s distress are positively associated with helping decisions would support existing empathy-based explanations of helping. Indeed, it has been proposed that helping a conspecific in danger results primarily from an evolutionarily preserved motivation to care for offspring in mammals, which is triggered by signals of distress and vulnerability, and is especially likely to occur *if* the helper is not under threat themselves (Preston, 2013). However, evidence in rodents indicates that, rather than conflicting, defensive responses may be *required* for helping and caregiving: for example, anxious rat mothers display enhanced maternal behavior after pharmacological activation of defensive brain circuits (Bosch, 2013; Bosch, 2011; Bosch et al., 2005), whereas mice bred to have low anxiety display significant defects in maternal behaviors (Sheleg et al., 2017); and helping behavior in rats is compromised following treatment with anxiolytic drugs that suppress defensive circuits (Ben-Ami Bartal et al., 2016). According to these animal findings, an alternative prediction to empathy-based accounts is that the neural representation of threat to the self would promote helping of others.

## Results

### Helping decisions did not vary based on threat imminence or threat level

**Figure 2.**
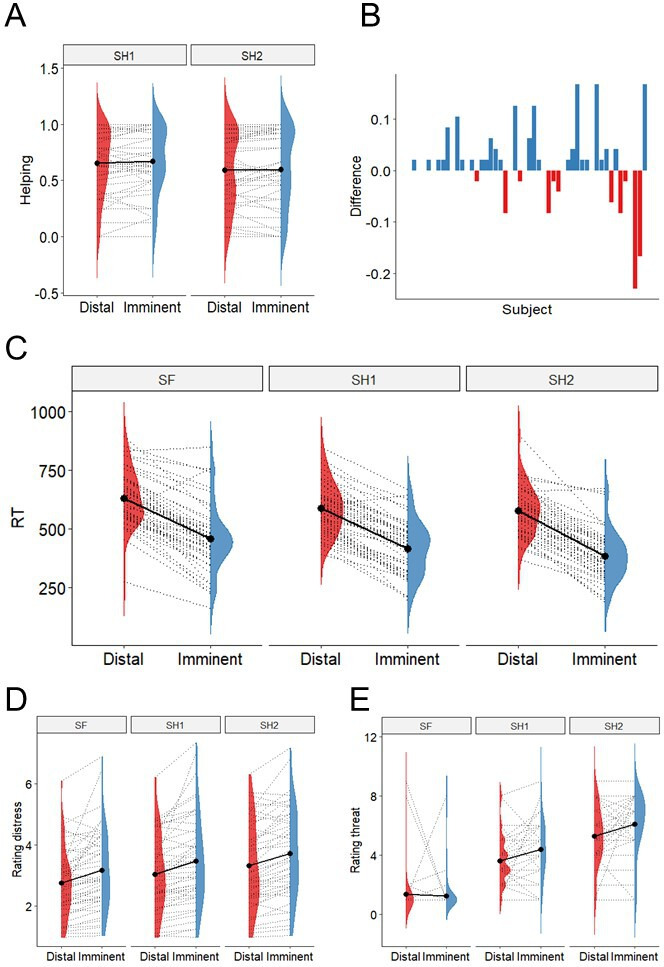
Behavioural results. (**A**). There was no evidence of differential helping during imminent and distal threats, nor during 1-shock and 2-shock trials. (**B**) Difference between proportion of helping in imminent and distal trials (y axis) across subjects (x axis); 27 participants helped more during imminent than distal threats, 11 helped more during distal, and 11 helped the same amount. (**C**) Responses were faster during imminent than distal trials across threat levels. (**D**) Participants rated the co-participant’s distress as higher during imminent than distal trials, and as progressively higher across the three threat levels. (**E**) Participants reported feeling more threatened when watching 2-shock cues (red circle), followed by 1-shock cues (yellow circle), and safe cues (green circle), and when watching cues signaling imminent than distal threat.

Using Generalized Linear Mixed Models (GLMMs), we found no significant effect of either threat imminence (β=0.017, se=0.014, t=1.24, p=0.218), threat level (β=−0.068, se=0.053, t=−1.282, p=0.205), nor a threat imminence*level interaction (β=−0.099, se=0.019, t=−0.497, p=0.622) on the percentage of helping decisions throughout the task (Figure 2A). Despite the lack of group-level effects of threat imminence (which were predicted based on previous work; Vieira et al., 2020), individual data showed that the number of participants helping more during imminent than distal threats was objectively higher (n=27) than those helping more during distal (n=11), or the same amount during imminent and distal (n=11) (Figure 2B). Note that these differences are descriptive, and no statistical inference was performed.

Previous work also suggested the impact of threat imminence on helping might vary based on empathic tendencies (Vieira et al., 2020). We thus checked whether there was an interaction between the empathic concern scale of the Interpersonal Reactivity Inventory (Davis, 1983) and threat imminence on helping. When empathic concern was added in the model, results showed no significant effects of threat imminence (β=0.018, se=0.014, t=1.249, p=0.215) and threat level (β=−0.050, se=0.051, t=−0.985, p=0.330), and no significant interaction between imminence and empathic concern (β=0.018, se=0.014, t=1.292, p=0.20). However, a significant association emerged between empathic concern and helping behavior (β=−0.101, se=0.041, t=−2.473, p=0.02), indicating those higher in empathic concern displayed less frequent helping behavior. It should be noted here that the impact of low statistical power cannot be discounted. Our previous study (Vieira et al., 2020) had a larger sample, and to detect a correlation between helping and empathic concern of the magnitude of that reported in that study (with 80% power) we would need at least 123 participants (based on calculations carried on in GPower 3.1.9.2).

Also, to account for the possibility that decisions varied throughout the experiment (e.g., participants helped more in the beginning than toward the end), we also performed a mixed effects logistic regression on single trial dichotomous responses (help or no help), including the trial number as a fixed effect. This analysis revealed no significant effects, indicating the individuals did not respond differently as time passed.

Finally, in line with previous work (Vieira et al., 2020), analysis of reaction times showed individuals made faster decisions during imminent versus distal trials (β=−173.30, se=12.09, t=−14.33, p<0.0001), and for shock versus safe trials (β[1 shock]=−43.26, se=12.76, t=−3.39, p=0.001; β[2 shock]=−53.35, se=13.43, t=−3.97, p=0.0002), with no significant threat imminence*level interaction (Figure 2C).

### Participants were sensitive to variations in the co-participant’s distress, threat imminence, and threat level (manipulation check)

After the scan, participants were asked to re-watch all clips of the co-participant during the scan, and rate the level of ‘discomfort, anxiety or uneasiness’ they thought *he* was experiencing in each clip. Of note, these clips were shown without the threat cues (see Figure 1B) that were also present during the scan, in order to isolate the response to distress and threat. Results showed participants rated the distress of the co-participant being significantly higher during imminent than distal clips (β=0.418, se=0.089, t=4.676, p<0.0001), and progressively higher across the three levels of threat (1 Sh: β=0.279, se=0.077, t=3.611, p=0.0005; 2 Sh: β=0.559, se=0.081, t=6.853, p<0.0001; reference class was safe). No significant threat level*imminence interaction was found (Figure 2F). These results suggest the video clips used in the scan successfully portrayed subtle variations in cues of distress by the co-participant.

Participants also presented isolated images of the threat cues used during the scanning task (namely, the green, yellow, and red circles, both in the distal and imminent positions; Figure 1B), and asked to rate how threatened they felt *themselves* when they saw those visual cues during the scan. Results showed participants rated threat stimuli as more threatening (1 Sh: β=2.255, se=0.297, t=7.598, p<0.0001; 2 Sh: β=3.92, se=0.297, t=13.89, p<0.0001; reference class was safe; Figure 2G). Additionally, imminent cues were rated as more threatening, but only for 1 shock and 2 shocks, and not for safe trials (imminence*1 Sh: β=0.894, se=0.420, t=2.129, p=0.034.; imminence*2 Sh: β=0.936, se=0.420, t=2.23, p=0.027).

### Neural responses

**Figure 3.**
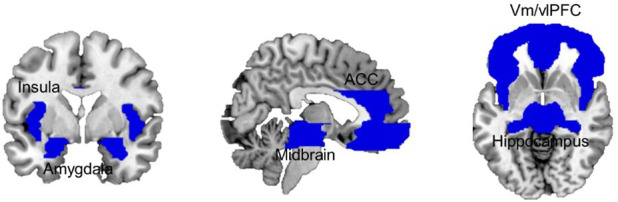
Combined ROI mask including bilateral ventral and lateral medial frontal cortex, dorsal ACC, insula, hippocampus, amygdala, and midbrain. ACC, anterior cingulate cortex; ROI, region of interest.

Analysis of brain responses focused on a liberally defined set of brain regions that integrate the brain’s defensive system, namely the vmPFC and vlPFC/IFG (Mobbs et al., 2009; Mobbs et al., 2010; Wendt et al., 2017), the hippocampus (Qi et al., 2018), the insula (Mobbs et al., 2010; Wendt et al., 2017), the ACC (Mobbs et al., 2009; Mobbs et al., 2010), the amygdala (Terburg et al., 2018; Mobbs et al., 2009), and the midbrain (Qi et al., 2018; Mobbs et al., 2009; Mobbs et al., 2010; Wendt et al., 2017; Mobbs et al., 2007; Figure 3). The full size of the brain mask used included 23,269 voxels (186,152 mm^3^).

#### Multivariate and univariate differentiation of threat imminence and level in the defensive circuitry

**Figure 4.**
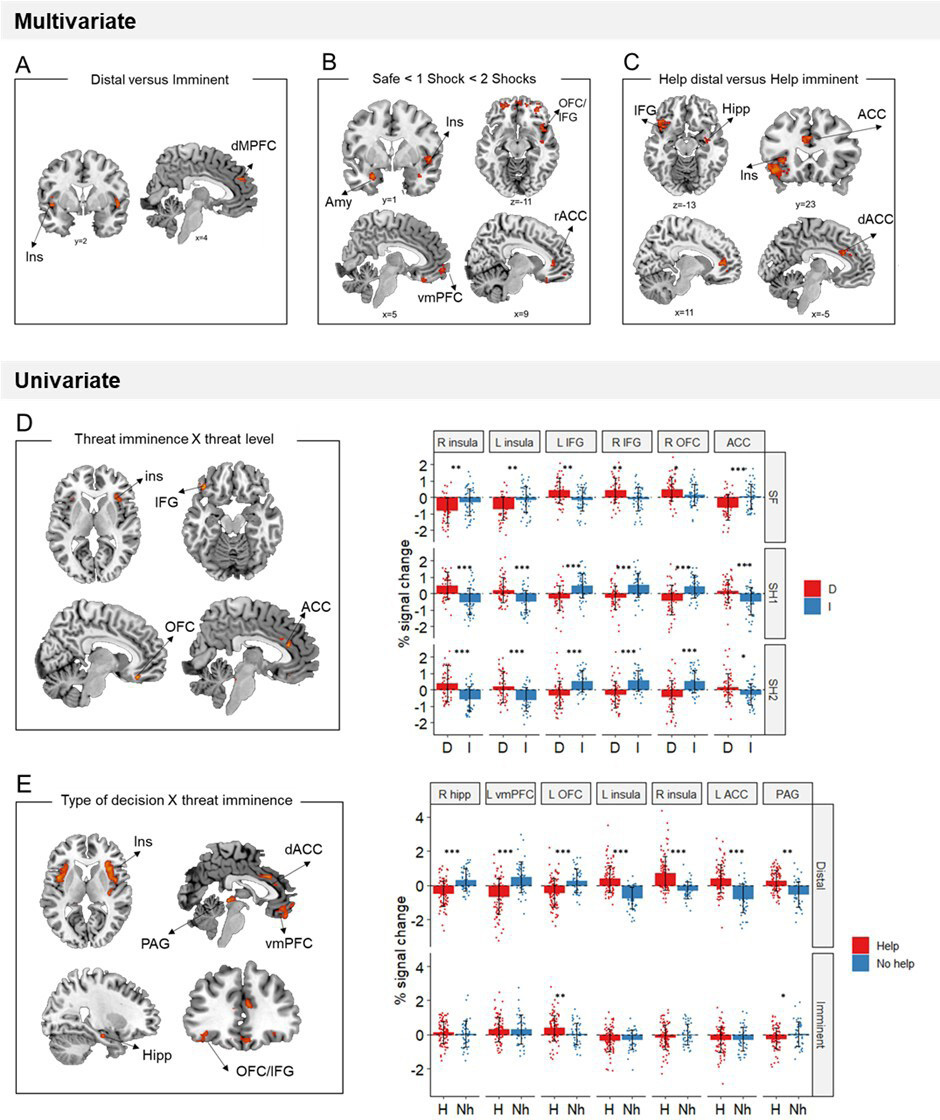
Multivariate and univariate fMRI results. (**A**) Local multivoxel activation patterns (identified by searchlight analysis) in the insula and dmPFC were distinguishable between distal and imminent threats, irrespective of helping decisions. (**B**) Local multivoxel activation patterns (identified by support vector regression) in the amygdala, insula, OFC/IFG, vmPFC, and ACC were linearly associated with varying threat level. (**C**) Local multivoxel activation patterns (identified by searchlight) in the insula, IFG, hippocampus, and ACC were distinguishable when making helping decisions under distal and imminent threat. (**D**) Clusters in the insula, IFG, OFC, and ACC displayed a significant threat imminence*threat level interaction. (**E**) Clusters in the insula, ACC, IFG/OFC, vmPFC, hippocampus, and PAG displayed a significant type of decision*threat imminence interaction. ACC, anterior cingulate cortex; AMY, amygdala; dmPFC, dorsomedial prefrontal cortex; Hipp, hippocampus; IFG, inferior frontal gyrus; Ins, insula; OFC, orbitofrontal cortex; PAG, periaqueductal gray; vmPFC, ventromedial prefrontal cortex. *p<0.05, **p<0.01, ***p<0.001.

We performed a support vector machine regression to identify sites in which activation patterns were linearly associated with increasing threat level (i.e., from safe, to 1 shock and 2 shocks). As predicted, results showed that throughout the defensive circuitry (i.e., amygdala, insula, ACC, hipoccampus, and regions within the orbitofrontal cortex) multivariate activation tracked with threat level (FWE<0.05, k>10; Figure 4B).

Additionally, to identify brain regions with differential average activation to the imminence of threatening stimuli specifically, we ran a univariate threat imminence (distal, imminent) by threat level (safe, 1 shock, and 2 shocks) ANOVA. Brain regions displaying a significant threat imminence by threat level interaction included the bilateral insula, OFC and IFG and ACC (Table 1; Figure 4D). In shock trials (both 1 shock and 2 shocks), the bilateral insula and ACC presented higher activation for distal compared to imminent threats, whereas the bilateral IFG and right OFC showed higher activation in imminent compared to distal threats (full ANOVA results in Appendix 1—table 1). These results were opposite to our predictions that brain regions previously implicated in reactive fear circuits (i.e., insula and ACC) would be more active during imminent threats, and regions implicated in cognitive fear circuits (i.e., IFG and OFC) during distal threats. Finally, using a Searchlight cross-classification algorithm (12-mm-radius sphere), we also identified brain sites in which multivariate patterns were distinguishable between distal and imminent threats. We found that multivariate patterns in the bilateral insula and dorsal medial prefrontal gyrus dissociated between distal and imminent threats (FWE<0.05, k>10; Figure 4A).

**Table 1.** Multivariate results based on threat imminence (distal—imminent) and level (safe, 1 shock, 2 shocks; FWE<0.05).

Searchlight distal versus Imminent					
	**R/L**	**k**	**x, y, z**	**T**	**BA**
Insula, superior temporal gyrus	R	40	46, –4, –8	7.43	22
Insula	L	17	–46, 2, –4	6.64	13
		18	–42, 4, 8	7.15	
Medial prefrontal cortex	R	20	10, 48, 28	7.13	9
**SVM regression SF – 1 SH – 2SH**					
Hippocampus	R	12	24, –10, –20	7.56	
Insula	R	25	42, –8, –8	8.34	13
Rolandic operculum	R	139	52, 0, 0	7.12	22, 47
Superior temporal gyrus, amygdala	R	11	38, 2, –24	7.73	
Amygdala	L	37	–24, 4, –24	7.96	
Rectus	R	12	6, 30, –24	7.07	11
Anterior cingulate	R	13	8, 38, 8	7.02	
Middle orbital frontal gyrus	R	42	32, 44, –14	7.32	11
Middle orbital frontal gyrus	L	11	–4, 48, –10	6.75	11
		44	–28, 54, –10	7.59	
		41	40, 56, –4	7.18	
Superior frontal orbital gyrus	R	19	16, 54, –14	6.78	10
Medial frontal gyrus	R	30	6, 58, –8	6.97	10
**Searchlight help during distal versus imminent threats**					
	R	29	36, –8, –16	8.04	
Hippocampus	R	21	50, 4, –2	7.01	22
Insula	R	23	32, 14, 14	7.18	13
Inferior frontal gyrus	L	207	–48, 20, –6	8.81	47, 38, 22, 13, 45
Mid cingulate, dorsal anterior cingulate	R	43	2, 22, 30	7.25	32, 9, 24, 6
Insula	L	45	–32, 26, 0	7.83	47, 13, 45
Anterior cingulate	R	53	10, 40, 8	7.92	32, 10

#### Greater engagement of reactive fear circuits led to helping

To test our prediction that higher engagement of reactive fear circuits would lead to helping, we performed a decision type (help, not help) by threat imminence (distal, imminent) ANOVA, and focused on brain regions displaying a significant interaction between the two (which would indicate activation differences when making decisions under distal and imminent threat). Of note, due to the reduced number of not helping trials, ‘no help’ decisions in this analysis included not only threat trials in which participants did not help, but also responses made in safe trials (details in Materials and methods). Results showed a significant interaction in the midbrain PAG, bilateral insula, right hippocampus, dorsal ACC, OFC, and vmPFC, which was driven by the distal condition (Table 2; Figure 4E; Appendix 1—table 2). Indeed, during distal threats, higher activation in the hippocampus, vmPFC, and OFC was followed by decisions not to help, whereas higher activation in the dACC, PAG, and insula led to helping decisions.

**Table 2.** Results of the univariate ANOVAS (FWE<0.05).

Univariate threat level*imminence interaction										
	**R/L**		**k**	**x, y, z**		**F**		**BA**		
Insula	R		768	38, 20, 6		28.14		13		
Insula	L		521	–30, 24, –4		20.58		13		
IFG, OFC	L		593	–40, 32, –16		23.72		11		
OFC	R		407	8, 32, –20		23.43		11		
ACC	R		278	4, 32, 20		18.29		24		
OFC, IFG	R		52	34, 40, –12		13.18		11, 47		
Type of decision*imminence interaction										
	**R/L**	**k**			**x, y, z**		**F**		**BA**	
Midbrain		60			2, –32, –4		16.85			
Insula	R	192			38, –16, –2		20.62		13, 47, 22, 44, 6, 45, 21	
Hippocampus	R	38			30, –14, –20		15.03			
Insula	L	595			−36, –8, –4		18.68		13, 22, 44, 6, 47, 45	
Dorsal anterior cingulate	L	151			–2, 14, 30		16.13		24, 6, 32, 5, 4	
Insula	R	748			30, 20, –8		18.18		47, 13, 22, 44, 6, 45, 21	
Inferior frontal/orbital gyrus	L	46			–34, 36, –10		11.47		11	
Rectus, ventral medial frontal gyrus	L	238			0, 46, –20		19.94		11, 10, 25	
										

One limitation of this analysis is that it conflated responses made during safe trials and no help responses. It could be argued that these represent fundamentally different types of decisions. To overcome this limitation, we also performed a parametric modulator analysis, in which we used a GLM that included a parametric modulator for decisions made in shock trials (0 if no help and 1 if helped). This analysis allowed us to model all threat imminence and level conditions, but only assign parametric modulators to those in which a subsequent help or no help decision was made (i.e., distal 1 shock, distal 2 shocks, imminent 1 shock, and imminent 2 shocks). The disadvantage of this approach is that only participants with at least 1 ‘no help trial’ trial per condition were included (N=28). Results of the parametric modulator analysis were consistent with those of the ANOVA (Table 3), in that we only found significant modulation of brain activation by subsequent decision during distal threats. Activation of bilateral insula was increased before helping decisions (distal 2 shocks), and activation of the vmPFC was increased before not helping decisions (distal 1 shock and distal 2 shocks).

**Table 3.** Results of the parametric modulation analysis.

Distal 1 shock					
	**R/L**	**k**	**x, y, z**	**T**	**BA**
(Neg) vmPFC, rectus	L	125	–4, 30, –22	6.55	11
**Distal 2 shocks**					
	**R/L**	**k**	**x, y, z**	**T**	**BA**
(Pos) Insula	R	110	44, 2, 4	5.76	13
(Pos) Insula	L	150	–38, 0, 8	5.56	13
(Neg) vmPFC, medial frontal orb	L	64	–4, 54, –12	5.13	11

**Figure 5.**
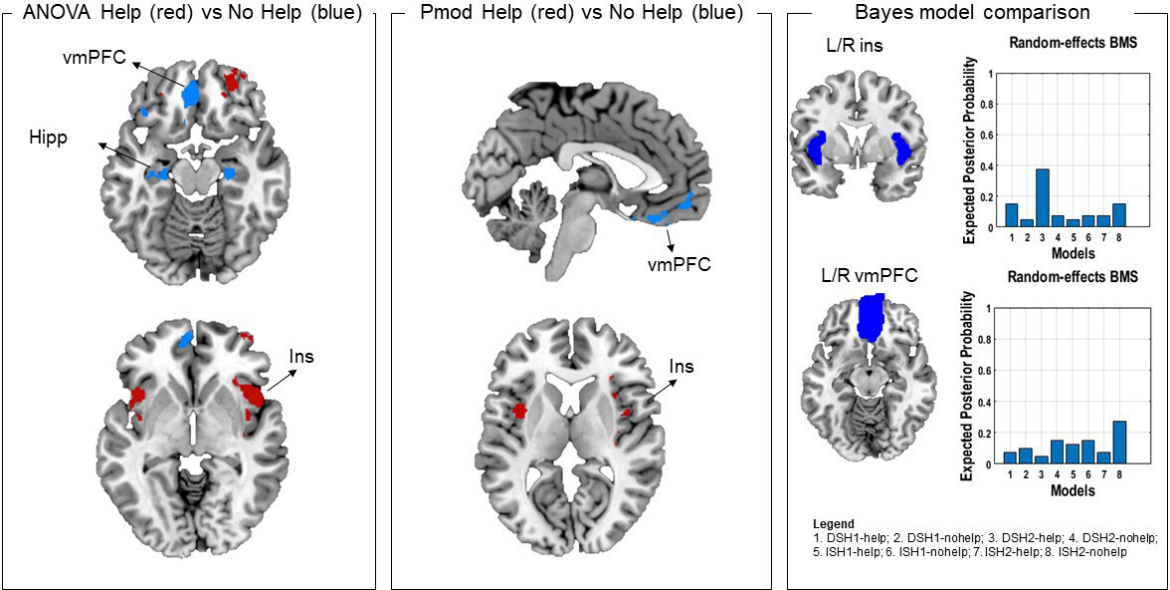
In the left and middle panels, comparison of ANOVA and parametric modulation activation maps. Since results of these two analyses suggested the effects were driven by the distal condition, here we selected the distal help versus no help (no help+safe) contrast from the ANOVA (left) and the help and no help maps from the parametric modulator regressors. Commonalities were found in the insula (activation associated with helping decisions) and in the vmPFC (associated with not helping decisions). Red denotes higher activation during help decisions, and blue denotes higher activation during not help decisions. In the right panel, results of the Bayesian model selection (BMS; following Bayesian first-level analysis). Resulting model evidence maps were thresholded at 0.75 (BF of approx. 8). ROI masks were then applied for model comparison. Results showed stronger evidence for help models for the insula, and no help models for the vmPFC, in line with the frequentist analyses. Note that results from the parametric modulation and Bayesian analysis are inherently noisier, given the smaller number of participants and trials. ROI, region of interest; vmPFC, ventromedial prefrontal cortex.

Following reviewer suggestions, we also estimated a first-level model that separated help, no help and safe decisions as a function of threat imminence and level, using a Bayesian approach (details in Materials and methods). The Bayesian first-level analysis was followed by model comparison (using random effects) on the target regions of interest (ROIs) that revealed significant results across the two frequentist analyses (i.e., insula and vmPFC). Using a probability threshold of 0.75 (corresponding to a BF of around 8) (Rosa et al., 2010), in the bilateral insula results indicated stronger evidence for the distal ‘help’ models (distal 1 shock and distal 2 shocks). In the bilateral vmPFC, results indicated stronger evidence across ‘no help’ models (distal 1 shock, distal 2 shocks, imminent 1 shock, and imminent 2 shocks) (Figure 5).

We additionally ran a Searchlight analysis to localize dissociable neural patterns guiding decisions under distal and imminent threats. Results showed that, prior to helping decisions, patterns of activation in the insula, hippocampus and dorsal cingulate were distinguishable between distal and imminent threats (FWE<0.05, k>10; Figure 4C).

#### Neural representations of threat promoted helping

**Figure 6.**
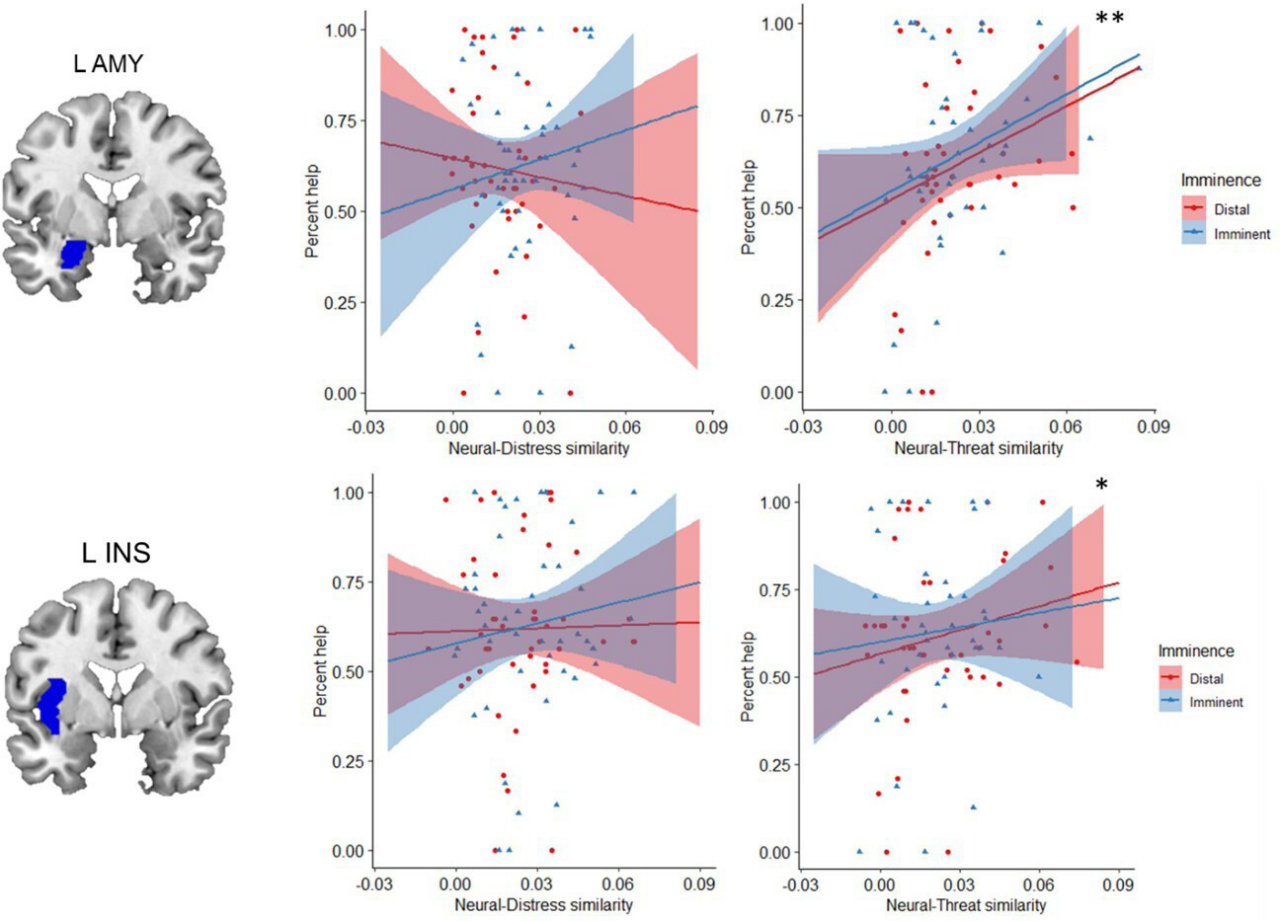
Regardless of threat imminence, the similarity between neural and threat RDMs in the left amygdala and insula predicted higher frequency of helping decisions. *p=0.047; **p=0.006. RDM, representational dissimilarity matrix.

One of our goals was to determine whether helping decisions were predominantly driven by the response to another person’s distress, by one’s own defensive state, or by both. To this end, we performed an ROI-based RSA (Diedrichsen and Kriegeskorte, 2017). This analysis was done separately for imminent and distal trials, and comprised three steps (see Figure 5 and Materials and methods for details). First, we computed neural representational dissimilarity matrices (RDMs) that reflected trial-by-trial variation in activation patterns throughout the scanning task. Second, we used post-scan ratings to construct behavioral RDMs that reflected, respectively, between-trial differences in perceived distress experienced by the co-participant, and between-trial differences in how threatened the participant felt themselves during the scan. Finally, in the third step, we estimated the second-order similarity between neural and behavioral RDMs. This similarity metric allowed us to assess, for each ROI, whether trial-by-trial multivoxel patterns during the scan primarily represented the co-participant’s distress, or the threat to oneself. Importantly, it allowed us to determine whether and to what degree neural representations of other’s distress and of threat to the self were associated with helping behavior. Based on the second-order similarity between neural and behavioral RDMs, we found no evidence for any of the ROIs that neural activity predominantly represented other’s distress or threat to self (Appendix 1—table 5). However, results showed that, regardless of threat imminence, the similarity between neural and threat RDMs in the left amygdala (β=4.41, se=1.35, t=3.27, p=0.006) and left insula (β=2.46, se=0.97, t=2.53, p=0.047) was positively associated with helping behavior (Figure 6; Appendix 1—table 3). In other words, the more strongly these brain regions, especially the amygdala, represented the threat to oneself, the more frequently the participant decided to help.

## Discussion

Our overarching goal with this study was to determine how one’s own defensive responses influence decisions to help others under threat. Our findings strongly suggested that neural circuits that coordinate fast avoidance responses from self-directed threats (reactive fear circuits) (Mobbs et al., 2020) also underlie the protection of others in dangerous situations. More, the extent to which key defensive regions (amygdala and insula) represent the threat to the self (and not others’ distress) predicts more frequent helping decisions.

### Reactive fear circuits promote both self- and -other defence

Previous work has shown that increased imminence of an other-directed threat facilitates helping behavior, suggesting that the same way threat imminence triggers active avoidance (i.e., fight or flight) from self-directed threats, it may promote defensive helping when others are under threat (Vieira et al., 2020). Here, we examined the neural basis of this effect. We found that multivoxel activation within the defensive circuitry (*i.e.,* amygdala, ACC, insula, hippocampus, and OFC) tracked with the level of threat. Also, we found dissociable local patterns of activation prior to helping as a function of threat imminence within the insula, hippocampus, and dorsal ACC. These findings are consistent with previous work highlighting the role of the hippocampus and dACC as key regions optimizing escape decisions within cognitive and reactive fear circuits, respectively (Qi et al., 2018). Importantly, we found that average activation within several regions of the defensive circuitry, namely the in the PAG, insula, hippocampus, dACC, OFC, and vmPFC, differed between trials leading up to helping or not helping decisions. Specifically, consistent with our predictions, activation in the insula, ACC, and PAG was higher before decisions to help, whereas activation in the hippocampus, vmPFC, and OFC was higher prior to decisions not to help. Therefore, although overall we did not find higher frequency of helping decisions under imminent than distal threat, our results suggest that greater engagement of reactive fear circuits facilitated helping behavior. Our failure to replicate prior behavioral effects of threat imminence could have been due to a difference in how participants were instructed (here, we instructed individuals to balance helping and not helping decisions) and/or statistical power (the previous study had a larger sample defined based on power calculations, N=98). The task instructions, in particular, used may have induced specific metacognitive processes that directly affected the behavioral decisions, and are virtually impossible to quantify. In any case, the present results are in line with prior findings in that they suggest that acute defensive states coordinated by reactive fear circuits promote helping under threat (Vieira et al., 2020). Of note, the differential activation of cognitive and reactive fear circuits based on the subsequent decision (help vs. not help) was only found when decisions were made under distal threat (i.e., when responses were prompted in the beginning of the trial). Also, contrary to our predictions, when comparing the average activation during distal and imminent threats independently of the decision, we found that the bilateral insula and ACC were more active during distal relative to imminent threats, whereas the bilateral IFG and OFC were more active during imminent threats. These results are opposite to those from prior studies that manipulated the imminence of self-directed threats (Mobbs et al., 2009; Wendt et al., 2017; Mobbs et al., 2007; Meyer et al., 2019). It is possible, however, that the disparity between our and previous findings is due to methodological reasons and does not reflect true differences in the processing of self- versus other-directed threats. For instance, in previous work only avoidance responses were made, whereas in our paradigm both help and no help decisions were possible (perhaps more analogous to an approach and avoidance option). Importantly, in our paradigm, every trial started with a distal threat (the circle was static on the left) that always evolved into an imminent threat (the circle would move to the right, and the shock would be administered at the end of the trial). This may have made the threat overly predictable (especially given the high number of trials required for fMRI), engaging reactive fear circuits to a greater extent during the distal phase of the trial.

Nonetheless, our results obtained in a paradigm wherein the threat was directed at another person (the co-participant) were overall consistent with previous fMRI research investigating neural responses to self-directed threats as a function of imminence, suggesting a parallel between responses to self- and other-directed threats. This self-other parallel has been demonstrated in other threat-related processes, such as learning (Olsson et al., 2020). Importantly, in line with previous demonstrations that acute defensive states may promote prosocial outcomes (Vieira et al., 2020), we found that greater engagement of reactive fear circuits may facilitate helping of others in a threatening situation.

### The neural representation of threat to the self predicts helping

To decide whether to help another person in a dangerous situation, one must consider not only their distress, but also the threat in the environment. Here, we determined how the representations of another’s distress and of threat guide behavior. To do so, we collected ratings of both the co-participant’s distress, and of how threatened the participant felt during the scan. Crucially, these ratings were obtained after the scan, allowing us to obtain a behavioral metric of how participants independently represented another person’s distress and the threat value of the situation. Although collecting these ratings during the scan would have provided a more direct measure of the neural representation of other’s distress and threat, it would have also have increased scanning time considerably and potentially compromised data quality as a result of fatigue. Performing these ratings after the scan was consistent with previous approaches (Parkinson et al., 2014) and enabled us to avoid explicitly priming participants to consider those distress and threat cues during the scan, which could have influenced their behavior and neural responses. Using behavioral representations of distress and threat, we assessed the extent to which each brain region in the defensive circuitry represented those cues, and its association with helping decisions. We found no indication that any of our ROIs predominantly represented the other’s distress or the threat to own self. However, our results showed that, regardless of threat imminence, the more the left amygdala and left insula represented the threat to oneself, the more participants decided to help.

The association between representation of threat and helping was particularly strong in the left amygdala. The amygdala has long been known to have a pivotal role in the acquisition and expression of defensive responses in mammals (Tovote et al., 2015). For instance, in both humans and rodents, it has been shown to coordinate switches between defensive states across through the communication between its basolateral nucleus (BLA) and oxytocin(OT)-sensitive neurons in the central amygdala (CeA) (Terburg et al., 2018). Our present results suggest that the amygdala’s role in defensive responding may also be relevant for helping behavior. This is consistent with previous work in animals. In rodents, CeA activation by OT not only enables the transition from freezing to fight-or-flight, but has also been shown to trigger offspring care behaviors in females (Rickenbacher et al., 2017), and to enhance maternal aggression (Bosch, 2013). It has additionally been demonstrated that the administration of benzodiazepines, drugs with a known effect on CeA (Griessner et al., 2021), impairs helping behavior in rats (Ben-Ami Bartal et al., 2016). Taken together with these reports, our findings in humans suggest that amygdala-mediated defensive processes may also enable the provision of care to others, here in the form of helping. Of note, our effects were restricted to the left amygdala, which is consistent with several others demonstrations of hemispheric specialization in amygdala function in emotional and pain processing (Allen et al., 2021; Baas et al., 2004; Blair et al., 2005; Butler et al., 2018; Young and Williams, 2010).

We found no evidence that the representation of other’s distress in any ROI was associated with helping, including in brain regions that have previously linked with empathy for pain and distress states (ACC and insula) (Bernhardt and Singer, 2012; Lockwood, 2016). Relatedly, our behavioral analysis revealed that, contrary to previous research (FeldmanHall et al., 2015; Hein et al., 2016), empathic concern was negatively associated with the percentage of helping decisions. These findings are at odds with prior accounts of altruistic responding, which suggest empathy for distress is the key proximal mechanism driving helping behavior (Batson et al., 1987; de Waal and Preston, 2017). Several explanations may account for lack of evidence of an association between neural representation of distress and helping in our data. One is that the behavioral ratings, which were collected after the scan, were not sensitive enough to reflect variations in perceived distress between clips of the confederate. However, this seems unlikely, given that we found significant effects of imminence and threat level on ratings of distress. Another possibility is that distress is mainly represented in brain regions not included in our analysis. The amygdala, insula and ACC would be prime candidate regions to represent another individual’s distress, in light of previous research on empathy (Fallon et al., 2020; Lamm et al., 2011; Marsh et al., 2014; OConnell et al., 2019), but we did not detect an association between the degree to which these regions represented distress and helping behavior. Other potential regions would be those previously implicated in mentalizing, such as the temporo-parietal junction (TPJ) (Lamm et al., 2011; Patel et al., 2019; Schurz et al., 2014). At an exploratory level, we repeated the RSA analysis within anatomical masks of the left and right TPJ, but did not find evidence that the representation of distress in these regions were related to helping behavior (see Appendix 1—table 4). Additional research at the whole brain level is necessary to further assess the representation of distress in the brain, and its impact on helping behavior under threat. In any case, our present results suggest that, even if perceiving distress/need in others to some extent triggers altruistic motivation (Preston, 2013), the ability to provide help may ultimately rely on the activation of circuitry implicated in self-defense.

In summary, our results point to a parallel between responses to self- and other-directed threats, and suggest that the engagement of reactive fear circuits facilitates helping of others. Importantly, we showed that the extent to which the amygdala represents the threat to self (and not other’s distress) predicts helping decisions. These results challenge the idea that empathy for distress is the only proximal mechanism motivating helping decisions, and that overriding self-defensive responses is necessary to help others under threat. Rather, in dangerous situations, one’s own response to the threatening event may enable defensive helping of others, possibly through the activation of neural mechanisms subserving both individual defense and offspring care in mammals.

## Materials and methods

This study has been pre-registered (https://osf.io/yvufn) and any deviations from the pre-registration are justified in the Appendix. Data and code will be made available at the OSF project page (https://osf.io/9cuva).

### Participants

Forty-nine healthy volunteers (M=24.29, SD=4.78) participated in the experiment. Participants were recruited via flyers posted on- and off-campus, and local online recruitment systems. All participants were right-handed, had normal or corrected-to-normal vision, and were screened for history of psychiatric or neurological diagnoses, current medication, brain injuries, and substance abuse. Participants provided informed consent prior to the experiment, and were compensated for their participation. This work was approved by the Swedish Ethics Board (Etikprövningsmyndigheten).

### fMRI helping under threat task

In each testing session, a participant and a confederate (henceforth, co-participant) were informed the experiment comprised two parts (only one of those parts involved an MRI scan), which would be randomly assigned to each one by flipping a coin. Participant and co-participant were then accompanied to separate testing rooms (the actual participant was taken to the MRI area) and did not interact again during the experiment (details about testing procedures, post-task questionnaires, and debriefing are available in the Appendix).

In the MRI, participants performed a task modified from previous work (Vieira et al., 2020) wherein they made trial-by-trial decisions about whether or not to help the co-participant avoid aversive electrical shocks to the wrist, at the risk of also being shocked (Figure 1A). Threat imminence was manipulated by varying the spatial position and movement of a visual cue signaling varying levels of threat on a computer screen. Respectively, a green circle signaled no threat (no shocks), a yellow circle signaled moderate threat (1 upcoming shock), and the red circle signaled high threat (2 upcoming shocks). In addition, a webcam feed of the co-participant was presented on the screen throughout the task. Unbeknownst to the participant, the video feed was in fact pre-recorded, and edited to select unique clips for each trial of the task.

Participants were informed that, throughout the experiment, they and the co-participant would see the same screen. Each trial started with a static cue on the left side of the screen (4 s), which then moved to the right (4 s). In shock trials, the co-participant would be administered an aversive shock to the wrist when the cue reached the right end of the screen, unless participants decided to help him. To decide whether they wanted to help the co-participant avoid the upcoming shock, participants made forced-choice responses by pressing 1 (Help) or 2 (Do not help) on an MRI-compatible button box as soon as the response slide was displayed (1.25–1.75 s). Responses were prompted sometimes in the beginning of the trial, when the visual cue was static on the left side (distal threat), and other times at the end of the trial, after the visual cue had moved to an endpoint on the right, and thus immediately before shock delivery (imminent threat). Of note, the time available to make a response was identical in distal and imminent conditions. Although in naturalistic settings higher threat imminence generally coincides with less time to make a decision, here we opted to dissociate imminence from decision for the sake of experimental control. Outcomes of participants’ decisions were as follows: if they chose not to help, the co-participant would always receive a shock; if they chose to help, there would be around 70% probability of both participant and co-participant receiving 1 shock (in moderate threat trials) or 2 shocks (high threat trials). Shocks were administered on the left ankle. Participants were instructed they should respond as quickly as possible. Also, to discourage missed responses, they were informed that a shock would be delivered to both participants (with 100% chance) whenever a response was not detected. Finally, to balance the number of helping and non-helping trials during the scanning session, participants were informed that they would have a pre-set number of times they could help on each run, and thus they should try to balance, per run, the number of times they helped and not helped. In reality, participants could help on as many trials as they wished. These task instructions may have affected overall helping performance as participants were asked to consider the overall balance of helping and not helping choices whenever making a new decision. Nonetheless, participants were still left with the decision whether to help or not on any given trial, allowing us to examine the corresponding brain activation. It is also important to point out that these instructions may have introduced additional metacognitive demands on the task. Yet, given the within-subject design, these demands are not expected to have introduced a systematic bias in the data. Shock administration always happened at the end of the trial, and participants were able to see the outcome of their decisions on the screen (i.e., the co-participant receiving or not receiving a shock; 4 s).

Safe trials followed an identical structure, with response slides presented at distal or imminent stages in relation to the end of the trial. However, participants were instructed that no shocks would be given and they should arbitrarily choose to press 1 or 2 when the response slide was displayed. It was made clear to them that their choice would have no consequences for them or the co-participant.

The task included 144 trials split into 8 functional runs (approx. 8 min). Each run comprised 18 trials, 9 distal and 9 imminent, and 6 of each threat level (resulting in 24 trials per condition, in total). Distal and imminent trials were presented in blocks, and the order of blocks was in each run. Within each block, safe, moderate threat, and high threat trials were randomized. The order of functional runs was randomized across participants. The task was programmed and delivered using E-prime 3.0 (Psychology Software Tools, Inc, https://www.pstnet.com).

### Ratings

After the scan, participants were taken to a different testing room and asked to complete a follow-up task. Here, all video clips showed during the scanning task were presented to participants, in random order. Participants were informed that these had been recorded during the scan, and that their task now was to, for each clip, rate the level of distress, anxiety or concern they perceived in the co-participant, on a 9-point scale. Participants also presented images of the visual cues at distal (left side of the screen) and imminent positions (right side of the screen), and asked to rate on a 9-point scale how threatened they felt during the scan, whenever they saw those images (Figure 1B). Ratings of distress and threat were presented in separate blocks, and the order was randomized.

### fMRI acquisition and preprocessing

Participants were scanned in a single session at the Stockholm University Brain Imaging Center (SUBIC), using a 3T Siemens scanner with a 64-channel head coil. First, a high-resolution T1-weighted anatomical scan was obtained (TR=2300 ms, TE=2.98 ms; FoV=256 mm, flip angle=9°, and 192 axial slices of 1 mm isovoxels), followed by 8 functional runs, of about 8 min each. Functional images were acquired with an echo-planar T2*-weighted imaging sequence with whole-brain coverage while participants performed the fMRI task (TR=1920 ms, TE=30 ms, FoV=192 mm, flip angle=70°, 62 interleaved slices of 2 mm isovoxels, and acceleration factor of 2).

Preprocessing of fMRI data was done using SPM12 (Wellcome Trust Centre for Neuroimaging; https://www.fil.ion.ucl.ac.uk), and included slice timing correction, realignment to the volume acquired immediately before the anatomical scan (i.e., the first image of the first functional sequence) using six-parameter rigid-body transformations (translation M=0.02 mm, min=−0.4, max=0.6; rotation M=0.0006 mm, min=0.02, max=0.008), coregistration with the structural data, normalization to standard space using the Montreal Neurological Institute (MNI) template with a voxel size of 2×2×2 mm^3^, and smoothing using a Gaussian kernel with an isotropic full-width-half-maximum of 4 mm (Gardumi et al., 2016; Hendriks et al., 2017). Finally, a high-pass filter with a cutoff of 128 s was applied to remove slow signal drifts.

### Statistical analysis

#### Behavioral data

Behavioral data was in general analyzed using GLMMs, an approach that accounts for variation in the dependent variable that is explained by random sampling of, for instance, participant or trial number (random effects), in addition to the independent variables (fixed effects). Mixed-effects approaches have further been proposed to increase the generalizability of research findings to other individuals and stimuli (Yarkoni, 2020).

Our main behavioral variable was helping behavior, which was operationalized as the percentage of helping responses throughout the task. We modeled helping percentage using GLMMs as a function of threat imminence, threat level, and imminence by threat interaction (fixed effects). The subject was added as a random effect, with random intercept and slope per threat imminence and level. In a separate model, we also added a threat imminence*level*empathic concern interaction, following previous indications that threat imminence may affect helping behavior more strongly in individuals with higher caregiving tendencies. To account for the possibility that behavior varied throughout the experiment, we also performed a mixed effects logistic regression on single trial dichotomous responses (help or no help), including the trial number as a fixed effect in addition to the other fixed effects. Finally, following recent recommendations to consider within-individual effect sizes (Grice et al., 2020), we also calculated the difference between number of helping decisions under imminence and distal threat, per individual.

Reaction times were averaged per condition, and analyzed in a GLMM with threat imminence, threat level, and imminence by level interaction as fixed effects, and the subject as a random effect (intercept and slope). Similarly, post-task ratings of other’s distress and threat to self were analyzed in a GLMM with threat imminence, threat level, and imminence by level interaction as fixed effects, and the subject as a random effect (intercept and slope).

#### Imaging data

##### First-level analysis

First-level analysis was performed in SPM12 and was based on the general linear model. Time-series of each voxel were normalized by dividing the signal intensity of a given voxel at each point by the mean signal intensity of that voxel for each run and multiplying it by 100. Resulting regression coefficients thus represent a percent signal change from the mean. Regressors were created by convolving the train of stimulus events with a canonical hemodynamic response function. Three different GLMs were estimated based on the goal of the analysis. For assessing differences based on threat imminence and level, six regressors of interest were modeled corresponding to the time window of the visual threat cue (distal safe, distal 1 shock, distal 2 shocks, imminent safe, imminent 1 shock, and imminent 2 shocks). These regressors were defined based on the position of the threat cue on the screen (static=distal; approaching=imminent), and not relative to when the participant made a decision. In addition, eight regressors of no interest were added in the model, corresponding to the time window of the response and the outcome, plus the six motion parameters estimated during realignment.

To assess differences in neural response based on the type of decision, another model was estimated with six regressors of interest: help distal 1 shock, help imminent 1 shock, help distal 2 shocks, help imminent 2 shocks, no help distal, and no help imminent. Here, distal and imminent refer specifically to when the decision was prompted in the trial. Of note, due to the reduced number of no helping trials for some participants, the ‘no help’ regressor included both not help decisions, and decisions made during safe trials, wherein no shocks were given. In addition, eight regressors of no interest (response, outcome, and six motion parameters) were added. Because of potential concerns in modelling no help and safe trials together, we additionally created a model that included regressors for distal and imminent events (per threat level) with a subsequent decision, and for each shock regressor (1 shock and 2 shocks) we added a parametric modulator to reflect the response subsequently made in that trial (0=no help, 1=help). The limitation of this approach is that only participants with at least 1 ‘no help trial’ trial per condition were included (N=28). Following reviewer advice, we also implemented a Bayesian approach (using Variational Bayes in SPM12) to obtain a first-level model that produced separate estimates for help, no help and safe trials, based on threat imminence and level. We then performed model comparison on ROI evidence maps thresholded at p>0.75 (following Rosa et al., 2010).

Finally, a fourth model was created to enable subsequent trial-by-trial RSA, wherein one regressor was estimated for each individual trial, modelling the time window of the threat cue.

##### Regions of interest

Given our focus on defensive brain circuitry, our analysis targeted pre-specified ROIs that were anatomically defined, including the left and right amygdala, left and right hippocampus, left and right insula, midbrain, left and right ACC, left and right vmPFC, and left and right vlPFC (Figure 3). ROIs were defined on the Wake Forest University (WFU) Pickatlas toolbox (http://www.fmri.wfubmc.edu/cms/software; Maldjian et al., 2003).

##### Multivoxel pattern analysis and support vector regression

Beta values derived from first-level analyses were used in multivariate analyses, including multivoxel pattern analysis (MVPA) searchlight, support vector regression (SVR), and RSA. Spatially distributed patterns of activation across voxels can reveal distinguishable neural responses between experimental conditions even in the absence of significant average activation differences in single voxels, making multivariate approaches more sensitive than conventional univariate analysis (Formisano et al., 2008).

To identify local activation patterns that distinguish between distal and imminent threats, we used an MVPA Searchlight implemented in The Decoding Toolbox (TDT). A spherical searchlight (radius 12 mm) was moved throughout each participant’s data and, at each searchlight center, a support vector machine algorithm was trained to discriminate activation patterns in response to distal and imminent threats. Training was done iteratively on each 7 functional runs and tested on the 8th (leave-one-out cross-validation). Resulting percentage score at each voxel for participant was calculated and displayed in individual accuracy maps. Accuracy maps were then analyzed at the group level in a one-sample t test implemented in SPM12. A similar approach was taken to the identification of local activation patterns that discriminated helping decisions during distal and imminent threats. Additionally, an SVR was used to identify local patterns that showed a continuous linear association with threat level (safe, 1 shock, and 2 shocks). As for the classification searchlight, individual accuracy maps were analyzed at the group level in a one-sample t test. Group results were thresholded at voxelwise FWE<0.05 and only clusters with more than 10 voxels were further considered.

##### Univariate analysis

We also performed GLM-based univariate analyses. We analyzed BOLD signal to the threat cues, regardless of decision, in a threat imminence (distal and imminent) by threat level (safe, 1 shock, and 2 shocks) repeated-measures ANOVA. We also analyzed BOLD signal to threat cues prior to the decision in a decision type (help and not help) by threat imminence (distal and imminent) ANOVA. Full results for these analyses are available in . Finally, we performed a One-sample t test on parametric modulator contrast images to identify brain regions wherein average activation during the threat was modulated by subsequent decision. Univariate analysis results were first thresholded at p<0.001 uncorrected. With this threshold, clusters with more than 10 voxels were significant with FWE-corrected p<0.05.

##### Representational similarity analysis

**Figure 7.**
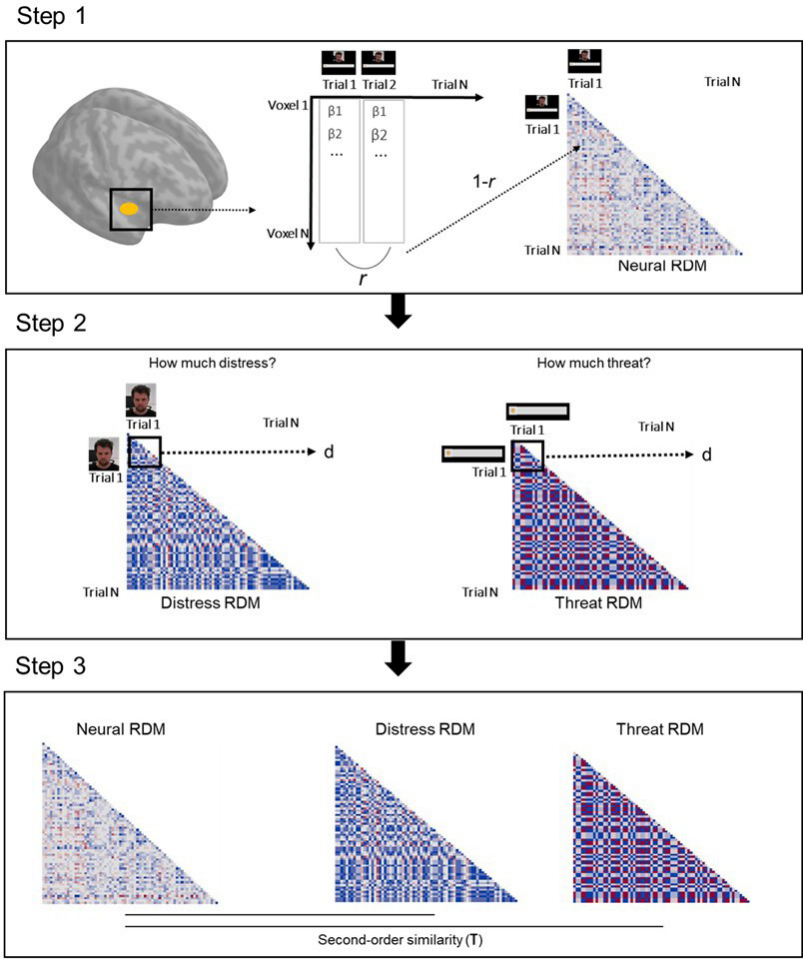
Schematic of the RSA pipeline. On step 1, we extracted the vector of trial-by-trial betas for each voxel in a given ROI. We then calculated the correlation (Pearson r) between all trial pairs. These correlation values were inverted (1−r) and used to create a trial-by-trial matrix, wherein each cell represents how correlated activation across all voxels of the ROI was in each trial pair (neural representational dissimilarity matrix, RDM). On step 2, post-scan ratings of the co-participant’s distress in each unique clip were used to construct a trial-by-trial matrix, wherein each cell contained the Euclidean distance between the rating of each pair of clips (distress RDM). A similar method was used with the ratings of threat to the participant (threat RDM). On step 3, the second-order similarity between the neural RDM and distress RDM, and between the neural RDM and threat RDM were calculated using a ranked correlation method (Kendall’s tau). ROI, region of interest; RSA, representational similarity analysis.

One of the goals of the study was to characterize representations of other’s distress and of threat to oneself by defensive regions, and determine its relation to helping behavior. RSA was done separately for imminent and distal trials, and comprised three steps (Figure 7). On the first step, we modeled each trial in the first-level analysis (in SPM12), in order to estimate one beta coefficient per trial. Then, for each ROI, we extracted betas from each voxel in each trial to estimate the correlation of beta-values (expressed in r values) between all voxels per trial. Resulting r values were used to construct a representational similarity matrix across all trials that reflects the correlation between all voxels in each trial. This matrix was then transformed (1−r) to reflect dissimilarity instead of similarity (RDM). On the second step, distress ratings provided after the scan on the unique video clips shown in each trial were used to construct a dissimilarity matrix that reflects the difference in perceived distress of the co-participant between trials (expressed in Euclidean distances). Post-scan threat level ratings were used in an identical manner to construct a dissimilarity matrix that reflects between-trial differences in how threatened the participant felt during the scan. Finally, on the third step, we estimated the second-order similarity (kendall’s τ) between neural and behavioral RDMs. In a nutshell, this similarity metric allowed us to assess, for each ROI, whether trial-by-trial multivoxel patterns during the scan represented the co-participant’s distress and the threat to oneself. Importantly, it allowed us to determine whether neural representations of other’s distress and of threat to oneself were associated with helping behavior. To do so, second-order similarity values were entered in a linear model predicting average helping percentage during the scan. Predictors in this model were the similarity between neural and threat RDM, the similarity between neural and distress RDM, as well as threat imminence. Thirteen linear models were estimated, one for each ROI (i.e., left and right amygdala, left and right hippocampus, left and right insula, midbrain, left and right ACC, left and right vmPFC, and left and right vlPFC). False discovery rate (FDR) correction was applied to adjust the p value of all coefficient estimates, across all 13 models. FDR-corrected p values below α=0.05 were considered significant. Beta value extraction was performed in Matlab, and all remaining steps and analyses of the RSA were performed through custom-made scripts in R (code available at https://osf.io/nb6cf/).

## Figures and Tables

**Appendix 1—figure 1.**
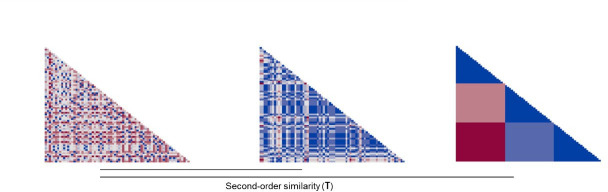
Example RDMs (Neural, Distress and Threat) sorted by threat level.

**Appendix 1—figure 2.**
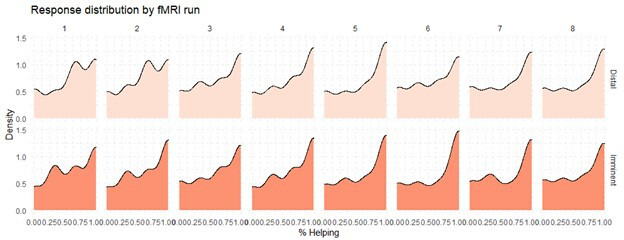
Response distributions per fMRI run.

**Appendix 1—figure 3.**
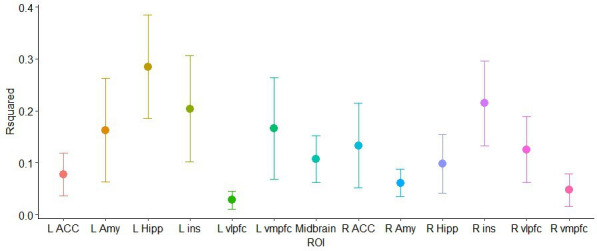
Cross-validation.

## Data Availability

This study has been pre-registered (https://osf.io/yvufn). Anonymized data, code, and materials used in the study are available on the OSF project page (osf.io/9cuva). The following dataset was generated: VieiraJB 2020Helping under threat - Part 2OSF9cuva

## Additional files

Transparent reporting form

## Appendix 1

### Experimental procedures

The experiment involved one session. Upon arrival, both participant and co-participant (i.e., confederate) were welcomed into the lab and told that the experiment comprised two parts and each one of them would only perform one. The parts were assigned through a fake coin flip to give the impression of randomization. Participant and co-participant were then accompanied to separate rooms to receive more detailed instructions.

Prior to entering the MRI, written informed consent was obtained, and electrodes for administration of electrical shocks were placed on participants’ left ankle. Participants were then given a short practice block on a computer, to allow them to familiarize with the timing of responses. After the practice, participants were taken into the MRI scanner room.

Once in the scanner, shock intensity was individually calibrated using a standard work-up procedure. Participants were asked to select an intensity level that was “not painful, but very uncomfortable” and that it should be something that “if they could avoid, they would rather avoid”. It was emphasized to participants that, during the experiment, they would be able to avoid the shock if they so desired.

Participants were then given written instructions for the first block of the task. They were informed they would see the co-participant via webcam and they would both be presented the same stimuli on the screen. The first block consisted of 3 trials (safe, 1 shock and 2 shocks), wherein the participant was simply required to pay attention to the screen; they would not be asked to make any responses and would not be given any shocks. The goal of this block was to allow participants to see the consequences of safe and threat trials to the co-participant. This was meant to discourage them from making “test responses” in the first trials of the actual task (for instance, decide not to help just to see whether the co-participant would indeed receive a shock).

Thereafter, participants were given written instructions for the rest of the task. They were explained that, on each trial, they would be asked whether they wanted to help the co-participant avoid the shock(s) or not, and what would be the outcomes of their decisions. Participants were also informed that: 1. the co-participant was not aware that shock administration was decided by them, 2. they should try to balance out the number of help and not help decisions, since there was a preset number of times they could help in each run, 3. they would not swap places with the co-participant afterwards, 4. they would not meet the co-participant again after finishing the experiment, and 5. their behavior during the task would not be observed nor filmed.

After the scan, participants were taken to another testing room to perform the ratings task. At the end, they were asked to fill out questionnaires, including post-tasks questions designed to assess whether the instructions and cover-story were believable (e.g., To what extent do you think the shocks were controlled by you? How authentic did the co-participant seem to you?). Lastly, they were fully debriefed.

### Post-task and questionnaire measures

After the fMRI helping under threat task and the ratings task, participants completed a series of questionnaires to assess individual differences in empathy and threat sensitivity, including the Interpersonal Reactivity Inventory (Davis, 1983), the Trait Fear Questionnaire (Kramer et al., 2020), the, and the Triarchic Psychopathy Measure (Patrick et al., 2009). Additionally, participants completed a series of post-task questions designed to assess the believability of the experiment.

### Univariate analysis

**Appendix 1—table 1.** Threat imminence X Threat level Anova.

Main effect of threat imminence						
	**R/L**	**k**	**x, y, z**	**F**	**BA**	
Insula	R	36	42, 2,–6	12.76	13	
Insula	L	113	–28, 12,–20	16.29	47	
IFG	R	1410	50, 20,–10	25.43	47	
vmPFC	L	334	–8, 32,–16	17.43	11, 25, 10	
OFC	L	37	–18, 56,–8	12.62	11	
**Main effect of threat level**						
	**R/L**	**k**	**x, y, z**	**F**	**BA**	
IFG	R	72	36, 24, 8	20.25	13, 45, 47, 44,	
vmPFC	L	443	0, 34,–16	27.35	10, 11, 32, 25, 9	
**Threat imminence*level**						
	**R/L**	**k**	**x, y, z**	**F**	**BA**	
Insula	R	768	38, 20, 6	28.14	13	
Insula	L	521	–30, 24,–4	20.58	13	
IFG, OFC	L	593	–40, 32,–16	23.72	11	
OFC	R	407	8, 32,–20	23.43	11	
ACC	R	278	4, 32, 20	18.29	24	
OFC, IFG	R	52	34, 40,–12	13.18	11, 47	

**Appendix 1—table 2.** Type of decision X Threat imminence Anova.

Main effect of type of decision						
	**R/L**	**k**	**x, y, z**	**F**	**BA**	
Hippocampus	L	128	−32,–18, –14	17.85		
Hippocampus	R	58	28,–16, –16	16.01		
Insula	L	638	–40, 8, 6	24.10	13, 47, 44, 45, 22	
Insula	R	949	46, 14,–2	30.43	13, 47, 44, 45, 22	
ACC	R	218	2, 22, 28	24.33	32, 6, 24, 8	
IFG	L	104	–38, 28,–14	12.38	21, 38, 47, 22	
vmPFC	L	768	0, 44,–18	24.74	11, 25, 10, 32	
OFC	R	129	36, 54,–14	14.50	11, 10	
**Main effect of threat imminence**						
	**R/L**	**k**	**x, y, z**	**F**	**BA**	
Insula	R	40	36,–20, 8	21.07	13	
		56	38, 8, 4	17.93	13	
		45	32, 20, 14	17.09	13, 45	
IFG	L	211	–50, 42,–8	29.37	47, 10	
vmPFC	L	447	–2, 54,–14	36.39	11	
**Type of decision*threat imminence**						
	**R/L**	**k**	**x, y, z**	**F**	**BA**	
Midbrain		60	2,–32, –4	16.85		
Insula	R	192	38,–16, –2	20.62	13, 47, 22, 44, 6, 45, 21	
Hippocampus	R	38	30,–14, –20	15.03		
Insula	L	595	−36,–8, –4	18.68	13, 22, 44, 6, 47, 45	
Dorsal anterior cingulate	L	151	–2, 14, 30	16.13	24, 6, 32, 5, 4	
Insula	R	748	30, 20,–8	18.18	47, 13, 22, 44, 6, 45, 21	
IFG/orbital gyrus	L	46	–34, 36,–10	11.47	11	
Ventral med frontal gyrus	L	238	0, 46,–20	19.94	11, 10, 25	

### RSA

**Appendix 1—table 3.** Estimates from models for each ROI, predicting helping percentage throughout the scan as a function of neural-distress similarity, neural-threat similarity, and threat imminence (all *p* values across all models were FDR-corrected).

	Estimate	Std. Error	t value	p (FDR-corrected)
**L amygdala**				
Intercept	0.536	0.054	9.823	<.00001
Threat	4.407	1.348	3.269	.**006***
Distress	–1.292	2.049	–0.630	.887
Imminence	0.040	0.055	0.731	.837
**L insula**				
Intercept	0.531	0.059	8.883	<.00001
Threat	2.461	0.974	2.525	.**047***
Distress	1.286	1.552	0.828	.837
Imminence	0.0129	0.052	0.246	.887
**L ACC**				
Intercept	0.649	0.057	11.257	<.00001
Threat	0.216	0.940	0.230	.887
Distress	–1.339	1.304	–1.026	.742
Imminence	0.018	0.054	0.348	.887
**L hippocampus**				
Intercept	0.620	0.067	9.233	<.00001
Threat	0.042	1.564	0.027	.978
Distress	0.104	1.642	0.063	.968
Imminence	0.017	0.054	0.324	.887
**Midbrain**				
Intercept	0.722	0.066	10.936	<.00001
Threat	–2.196	1.694	–1.296	.573
Distress	–2.622	1.753	–1.495	.451
Imminence	0.040	0.055	0.736	.837
**L vmPFC**				
Intercept	0.589	0.059	9.842	<.00001
Threat	0.752	1.010	0.744	.837
Distress	0.627	1.495	0.419	.887
Imminence	0.017	0.054	0.316	.887
**L vlPFC**				
Intercept	0.589	0.059	9.842	<.00001
Threat	0.752	1.010	0.744	.837
Distress	0.627	1.495	0.419	.887
Imminence	0.017	0.054	0.316	.887
**R amygdala**				
Intercept	0.695	0.062	11.170	<.00001
Threat	–1.651	1.584	–1.042	.742
Distress	–1.319	1.411	–0.935	.797
Imminence	0.017	0.053	0.332	.887
**R insula**				
Intercept	0.603	0.060	10.015	<.00001
Threat	0.436	0.976	0.447	.887
Distress	0.189	1.676	0.113	.946
Imminence	0.020	0.054	0.376	.887
**R ACC**				
Intercept	0.541	0.060	8.883	<.00001
Threat	1.781	1.279	1.392	.512
Distress	1.617	1.456	1.110	.739
Imminence	0.014	0.053	0.276	.887
**R hippocampus**				
Intercept	0.677	0.063	10.677	<.00001
Threat	–1.451	1.432	–1.012	.742
Distress	–0.704	1.350	–0.521	.887
Imminence	0.017	0.054	0.326	.887
**R vmPFC**				
Intercept	0.612	0.058	10.490	<.00001
Threat	0.933	1.055	0.884	.820
Distress	–0.467	1.345	–0.347	.887
Imminence	0.012	0.047	0.272	.887
**R vlPFC**				
Intercept	0.603	0.066	9.134	<.00001
Threat	–0.242	1.405	–0.172	.916
Distress	1.118	1.670	0.669	.876
Imminence	0.0207	0.054	0.381	.887

**Appendix 1—table 4.** Exploratory examination of association between neural-distress and neural-threat similarity, and helping in the temporo-parietal junction (TPJ) (anatomically defined ROI based on aal, including supramarginal and angula gyri). Estimates from linear models for left and right TPJ.

	Estimate	Std. Error	t value	p
**L TPJ**				
Intercept	0.552	0.051	10.756	<.00001
Threat	1.669	0.967	1.725	0.088
Distress	1.103	1.492	0.739	0.462
Imminence	0.014	0.053	0.269	0.789
**R TPJ**				
Intercept	0.559	0.057	9.859	<.00001
Threat	1.407	0.842	1.671	0.098
Distress	0.585	1.491	0.392	0.696
Imminence	0.018	0.054	0.339	0.735

**Appendix 1—table 5.** Comparison of Neural-Distress similarity, and Neural-Threat similarity, for distal and imminent threats, in each ROI.

	Distal	Imminent
L Acc	z=–0.039, *P*=0.969	z=–0.028, *P*=0.978
L Amy	z=–0.047, *P*=0.963	z=0.014, *P*=0.989
L Insula	z=–0.003, *P*=0.997	z=0.0064 p=0.995
L Vlpfc	z=–0.073, *P*=0.941	z=–0.036, *P*=0.971
L Vmpfc	z=–0.029, *P*=0.977	z=–0.036, *P*=0.971
L Hippocampus	z=0.001, *P*=0.999	z=0.002, *P*=0.998
Midbrain	z=–0.004, *P*=0.997	z=0.026, *P*=0.979
R Acc	z=–0.030, *P*=0.976	z=–0.005, *P*=0.996
R Amy	z=0.004, *P*=0.997	z=0.004, *P*=0.997
R Insula	z=–0.061, *P*=0.951	z=–0.039, *P*=0.969
R Vlpfc	z=–0.005, *P*=0.996	z=–0.027, *P*=0.978
R Vmpfc	z=0.002, *P*=0.998	z=–0.028, *P*=0.978
R Hippocampus	z=–0.010, *P*=0.992	z=0.010, *P*=0.992

### Cross-validation

Sample size was limited based on resources and no power analysis was performed. The number of trials was selected to guarantee a balance between having a reasonable number of data points per condition, and keeping the length of the scan within limits that were manageable for participants. The number of trials we used (144) was in fact higher than that of previous work that implemented similar RSA approaches with neural and behavioural data (e.g., Parkinson et al., 2014, *J Neurosci*; Kim et al., 2022, *NeuroImage*). To maximize generalizability, we have nonetheless followed the suggestion of one of the manuscript reviewers and implemented a cross-validation procedure for the RSA analysis.

Our RSA analysis comprised several steps:

1. Computing the initial voxel by trial matrix (which contains beta values for each voxel in each trial of the experiment).
2. Calculating the neural RDM (a trial x trial dissimilarity matrix in which each cell represents the correlation across all voxels of the ROI between pairs of trials).
3. Calculating second-order correlations between neural RDMs and behavioral RDMs, in which assessed the association between the neural activation pattern in each ROI and two behavioral models (provided by the distress and threat ratings),
4. And finally, predicting helping behavior in a linear model based on two predictors: the correlation between neural and distress RDM and the correlation between neural and threat RDM.
  The main goal of cross-validation is to guide the selection of the best fitting model. We thus used cross-validation on point 4 above, since in the previous points no model estimation or comparison was performed. For each ROI, we ran a 10-fold cross validation (between 5 and 10 folds are typically recommended) on the linear model predicting percentage of helping responses as a function of neural similarity with distress ratings, neural similarity with threat ratings, and threat imminence.

Resulting R squares per ROI are shown in the graph below, followed by a table with the full results (root mean squared error, RMSE, mean absolute error, MAE, and corresponding SDs):

### K-fold cross validation results

**Table:**

ROI	Rsquared	RsquaredSD	RMSE	RMSESD	MAE	MAESD
L Amy	0.163	0.200	0.237	0.047	0.178	0.047
L ins	0.204	0.206	0.245	0.036	0.187	0.027
L ACC	0.077	0.083	0.246	0.058	0.188	0.046
Midbrain	0.107	0.090	0.246	0.064	0.195	0.046
L Hipp	0.285	0.200	0.253	0.062	0.192	0.042
L vmpfc	0.166	0.195	0.246	0.056	0.190	0.039
L vlpfc	0.028	0.035	0.252	0.046	0.191	0.031
R Amy	0.061	0.053	0.247	0.055	0.186	0.040
R ins	0.215	0.164	0.254	0.051	0.193	0.034
R ACC	0.133	0.163	0.248	0.042	0.187	0.034
R Hipp	0.098	0.114	0.250	0.067	0.191	0.047
R vmpfc	0.047	0.062	0.251	0.036	0.189	0.034
R vlpfc	0.125	0.127	0.249	0.062	0.193	0.050

It should be noted that cross validation is especially valuable to guide subsequent model comparison and selection. This was not the goal of our linear model, which aimed at performing statistical inference on the predictive power of each of the predictors. Therefore, in the present case, the interpretability of the cross-validation procedure is limited.

### Deviations from pre-registration

A pre-registration for this study can be found at. Some changes were implemented in relation to the pre-registration, namely:

1. We restricted our analysis to ROIs for which we had predictions, instead of reporting whole-brain analysis. Our data, including the whole-brain maps, will be available on OSF.
2. Correction for multiple comparisons in the analysis of fMRI data was done via Family-Wise Error (FWE) and not False-Discovery Rate (FDR). FWE was deemed a more conservative approach, and adequate to our focus on regions-of-interest.
3. Group results for MVPA searchlight analyses were done with t-test and not permutations. This alteration was in line with recommended procedures (Hebart et al., 2014). Median maps were assessed to confirm they were in agreement with the parametric results.
4. Out of 49 participants, only one participant stated categorically not believing in the cover story. We deemed it to be a more conservative approach to keep this participant in the analysis, especially since it was unclear at what point of the experiment they started to have doubts.
5. We pre-registered an exploratory whole-brain RSA Searchlight analysis that is still ongoing, and is thus not included in the present manuscript.
