# Supplementary material for: Neural defensive circuits underlie helping under threat in humans
Source: eLife. 2022 Oct 25;11:e78162. doi: 10.7554/eLife.78162 (PMC9596154; doi:10.7554/eLife.78162)
Supplement: Peer review history

# Editor report: Editor's evaluation

**DOI:** 10.7554/eLife.78162.sa0

**Authors:** Alexander Shackman

**Affiliations:** University of Maryland United States

This work fills an important theoretical gap regarding the role of potential threats to the self in prosocial helping. Much of the prevailing knowledge about the motivations for prosocial behavior focuses on the distress of the conspecific-in-need. Leveraging animal research, the authors hypothesize that defensive neural circuitry may stimulate prosocial helping under threat, specifically that "cognitive" fear circuits would respond to more distal threats whereas "reactive" fear circuits would respond to imminent threats. In addition to examining helping behavior under conditions of threat to self, the authors included representational similarity analyses of neuroimaging data, examining how overlapping representations of self and other distress related to helping behavior. The potential to challenge existing empathy accounts of prosocial helping is intriguing and worthy of interrogation.

# Decision letter

**DOI:** 10.7554/eLife.78162.sa1

**Affiliations:** University of Maryland United States

Our editorial process produces two outputs: (i) public reviews designed to be posted alongside the preprint for the benefit of readers; (ii) feedback on the manuscript for the authors, including requests for revisions, shown below. We also include an acceptance summary that explains what the editors found interesting or important about the work.

**Decision letter after peer review:**

Thank you for submitting your article "Neural defensive circuits underlie helping under threat in humans" for consideration by *eLife*.

Your article has been reviewed by 2 peer Reviewers, and the evaluation has been overseen by Drs. Shackman (Reviewing Editor) and Roiser (Senior Editor).

The Reviewers have discussed their reviews with one another and the Reviewing Editor, who drafted this summary.

The Reviewers noted several strengths of the manuscript, including:

– The theoretical basis for this work is sound and the authors attempt to answer a question of broad interest.

– The questions the authors set out to investigate are interesting and original. Understanding the role of defensive neural circuitry in helping decisions is of interest to fields of neuroscience, ecology, and psychology.

– This topic of this paper will be of interest to readers in the field of decision–making under threat, fear and anxiety, defensive responses, and altruistic behaviors. With an ecologically inspired task design, the authors looked into the relationship between self–directed defensive mechanism and the action of helping others, and shed lights on the brain regions and neural circuits driving these behavioral dynamics.

– Conceptually, this paper taps into the dynamics between altruistic behaviors and defensive responses to simulated ecological threat in humans, which is substantially relevant but is to date a rare breed. This innovation helps advance the understanding of the intersection. It also builds strongly upon prior human/animal research, where the "cognitive fear" and "reactive fear" circuits on the threat imminence continuum has been established.

– The inclusion of threat imminence and threat value increase the contribution of this work to understanding how helping decisions vary as a function of threat features.

– The authors preregistered the study analyses and hypotheses.

Nevertheless, several substantial concerns considerably dampened enthusiasm and there was a consensus that additional data and major revisions would be required.

As Reviewer #1 noted, "The interpretation of these data is hindered by a number of technical and conceptual limitations with data analysis and experimental design. Most of the data analysis concerns can be addressed, but conceptual issues with the experimental design substantially limit the conclusions that can be drawn from the current study." S/he also emphasized that the study "requires essential additional data to support the central claims. As [both Reviewers] noted, the collapsing of "no help" choices makes it difficult for the authors to substantiate their primary claims about neural engagement in response to helping versus not helping. I also raised concerns about the second central claim, which is the argument that the RSA results demonstrate a unique representation of one's own threat, but not the other's distress. Without additional data addressing these two concerns, I am not confident the claims are substantiated. I have enthusiasm for this work…but these are pretty big" concerns.

As Reviewer #2 noted, "My concerns overlap with those of Reviewer #1. After reading [his/her] comments and the paper again, I agree that the authors would need to conduct additional control experiments and analysis to address the major limitations."

– Instructions/New Data

R1. The authors explicitly instructed participants that they "would have a pre–set number of times they could help on each run, and thus they should try to balance, per run, the number of times they helped and not helped".

R1. These instructions undermine most of the study conclusions. The verbal instruction to balance helping and non–helping behavior introduces an auxiliary constraint into the task, likely inducing metacognitive processes in participants via which they monitor their behavior to ensure that they satisfy the constraint. It is unclear how such metacognitive processes would alter neural activity during the task, making it difficult to distinguish whether the observed BOLD signal is involved in decision making or is also influenced by metacognition. The data indicate that most subjects heeded these instructions: Figure 2B illustrates that most subjects maintained differences of less than 10% in helping vs. non–helping behavior and approximately half maintained differences of less than 5%, suggesting that most subjects attempted to balance helping and non–helping decisions.

– RSA Analyses

R1. Although this study has strengths in principle, the weaknesses of this work result in an inability to support the conclusions the authors attempt to make. One of the stated goals of this work is to determine how the neural representations of self threat and other's distress are associated with helping behavior. To dissociate representations of threat and other's distress within the defensive circuitry would require the authors to show that the neural representations of threat are separable in voxel space from the neural representations of other's distress.

R1. The authors do not explicitly show that the two kinds of neural representations are dissociable or that neural representations are sufficiently stable within conditions to be used in an RSA. One can imagine that representational drift across trials could lead to high variance of neural representations within a single condition, leading to low similarity scores within that condition and therefore deflated second–order similarity scores. To show that the neural representations of self threat and other's distress are dissociable, the authors would need to first determine the distributions of these two kinds of representations in conditions where subjects are faced only with a threat to themselves or only with the distress of another. This could be done, for example, by continuing to image while subjects rated self threat and other's distress and extracting the distribution of voxel patterns that are correlated with these ratings; then, methods from signal detection theory could be used to determine if the two distributions are separable.

R1. The authors assume that when the threat and conspecific in need are presented simultaneously the neural representations in these contexts will be a linear combination of the separate representations for self threat and other's distress. Evidence from other domains suggests that may be unlikely; for example, in olfaction, sensory representations of mixtures of two odors are rarely a linear combination of the individual sensory representations of each odor presented separately.

R1. These conceptual issues with the RSA hinder its interpretation here and make it difficult to accept the authors' interpretation that "neural representations of threat promoted helping'.

– Data Pooling/Muddling

R1. In the section "Greater engagement of reactive fear circuits led to helping", the authors pool imaging data from trials on which a "no help" decision was made with imaging data from safe trials. A decision under threat to help a conspecific and an arbitrary decision with no consequences for either the subject or conspecific should involve different neural mechanisms, so there is no clear justification for pooling the data from these two conditions. Pooling data from these two conditions makes it impossible to determine whether the results of the ANOVA provide sufficient evidence for their conclusion that "greater engagement of reactive fear circuits led to helping'.

R2. As mentioned in line 227, because of a reduced number of "no help" decision trials in the conditions where threat is present, the authors used trials from the safe condition in conjunction to form the regressor. This might reflect an "inflation" in "help" decisions, possibly due to the nature of the experimental environment and task design [See comments regarding 'instructions']. This could influence the interpretation of the behavioral results – for example, there was no significant difference between the "help" ration in distal and imminent threat conditions, and that could be due to the saturated "helping" responses (line 798, Figure 2A). This is also potentially problematic for the imaging analysis, since there has been research showing that safety signals are implicated in the vmPFC regions, which overlap with the key ROIs listed in this research. The "no help" trials under safety would not be homogenous to the "no help" trials under threat.

R2. Both in the Results and Discussion sections (line 300 – 314), the authors mentioned that the average activation patterns for imminent and distal threats were contrary to previous research of similar nature. One possible explanation the paper might have overlooked is that the analysis would be done without the independence of "help/no help" decisions. In the previous research the paper cited, there was only one possible choice when under threat. However, in the current study, "help" resembles an approach choice, while "no help" an avoidance choice (and possibly safety signal). combining them would confound the results.

R1. The authors should re–run the analysis without pooling the safe and no–help conditions. If the authors are worried about a lack of data on ``no help' decisions, they could take a Bayesian approach to the analysis since Bayesian models are non–asymptotic and therefore do not make the same demands on sample size as frequentist approaches. Summary statistics for the distribution of no–help decisions should be included.

R2. The authors could consider separating the "help" and "no help" trials in a more stringent manner, or create some control scenarios where only one action is possible;

R2. The study would benefit from some adjustment to the paradigm itself – to reduce the "inflation" in helping, and increase the effectiveness of different levels of threat;

# Reply: Author response

**DOI:** 10.7554/eLife.78162.sa2

– Instructions/New Data

R1. The authors explicitly instructed participants that they "would have a pre–set number of times they could help on each run, and thus they should try to balance, per run, the number of times they helped and not helped".

R1. These instructions undermine most of the study conclusions. The verbal instruction to balance helping and non–helping behavior introduces an auxiliary constraint into the task, likely inducing metacognitive processes in participants via which they monitor their behavior to ensure that they satisfy the constraint. It is unclear how such metacognitive processes would alter neural activity during the task, making it difficult to distinguish whether the observed BOLD signal is involved in decision making or is also influenced by metacognition. The data indicate that most subjects heeded these instructions: Figure 2B illustrates that most subjects maintained differences of less than 10% in helping vs. non–helping behavior and approximately half maintained differences of less than 5%, suggesting that most subjects attempted to balance helping and non–helping decisions.

We thank the reviewer for their comment, and realize that our rationale for using these instructions was not clear. We disagree that the “instructions undermine most of the study conclusions”. The implementation of these instructions was motivated by our results in a previous study using a similar paradigm (Vieira et al., 2020, Proc B), in which we found markedly skewed response distributions caused by the high rate of helping. We also found evidence of this during piloting for the current fMRI study, in which this would be especially problematic because we needed participants to decide not to help in at least some trials so we could analyse the corresponding brain responses. Therefore, we (falsely) informed participants they would need to balance out the number of help and not help responses. We were fully aware that the total helping percentage throughout the task could be affected by the instructions, as the total number of helping responses would not necessarily reflect the number of times participants would like to have helped. But importantly, at the trial level, participants were still left with the decision of whether to help or not, and thus the corresponding brain response leading to the decision in each trial remained informative.

We agree with the reviewer that the verbal instruction could have induced metacognitive processes, but also believe that, based on participants’ subjective reports at the end of the study, a task of this nature will always introduce metacognitive processes over which we have no experimental control. The influence of such metacognitive processes would have been similar across conditions (given our within-subjects design), and not likely to introduce systematic bias in the results. The most crucial aspect, in our opinion, is that regardless of the experiment’s constraints, participants were still left with a trial-by-trial decision of whether to help or not.

Despite our confidence in the task instructions, we fully agree with the reviewer that more information is needed to justify their rationale and potential implications for the study conclusions. We therefore edited the manuscript as follows:

P. 15: “Our failure to replicate prior behavioural effects of threat imminence could have been due to a difference in how participants were instructed (here, we instructed individuals to balance helping and not helping decisions) and/or…”.

P. 22: “to balance the number of helping and non-helping trials during the scanning session, participants were informed that they would have a pre-set number of times they could help on each run, and thus they should try to balance, per run, the number of times they helped and not helped (…) Although these task instructions may have affected overall helping performance, at the trial level, participants were still left with the decision whether to help or not on any given trial, allowing us to examine the corresponding brain activation. It is also important to point out that these instructions may have introduced additional metacognitive demands on the task. Yet, given the within-subject design, these demands are not expected to have introduced a systematic bias in the data”.

– RSA Analyses

R1. Although this study has strengths in principle, the weaknesses of this work result in an inability to support the conclusions the authors attempt to make. One of the stated goals of this work is to determine how the neural representations of self threat and other's distress are associated with helping behavior. To dissociate representations of threat and other's distress within the defensive circuitry would require the authors to show that the neural representations of threat are separable in voxel space from the neural representations of other's distress.

We appreciate the reviewer’s point and agree that this is a key aspect we need to clarify. Our goal, as the reviewer mentioned, was to determine whether the neural representation of self threat and other's distress in specific brain regions was associated with helping behaviour. It was not to localize representations of distress and threat in voxel space within those regions. The localization of voxels exclusively representing distress or threat would not have answered our question, which was specifically related to how representation of a certain type of information across the entire ROI was associated with helping. In fact, based on previous studies we would expect several of the used ROIs to represent both self-threat and other’s distress (e.g., insula and ACC). Overlapping voxels in such regions could represent both other’s distress and threat to self, but only the representation of threat be correlated with helping across the whole ROI (as we found).

To make our objective clearer, we edited the introduction in the following way:

P. 6: “To dissociate the role of representations of threat and of other’s distress on helping behaviour, after the scan we asked participants to rate the degree of distress experienced by the co-participant in each clip showed during the scan; also, participants rated how threatened they felt themselves when they saw the visual threat cues during the scan. These ratings were used as behavioural models in an ROI-based representational similarity analysis (RSA; see Materials and methods) that identified neural representations of other’s distress and of threat to the self, and determined their association with helping behaviour. It should be noted that our goal was specifically related to the link between neural representations of distress and threat, and helping behaviour, and not to the dissociation of representations of distress and threat in the brain. This, however, would be a highly interesting question to examine in future research.”

Also, to provide additional information regarding the extent to which activity in our ROIs was predominantly correlated with the representation of self threat or other’s distress we compared the second-order similarity between the neural, threat and distress RDMs. In other words, we checked whether trial-by-trial neural activity in each ROI was differently associated with ratings of other’s distress or of self threat. These comparisons showed that, across all ROIs examined, there was no evidence that other’s distress or threat to self were predominantly represented. We now report this in the manuscript:

P. 13: “Based on the second-order similarity between neural and behavioural RDMs, we found no evidence for any of the ROIs that neural activity predominantly represented other’s distress or threat to self (Table S5)”.

We provide the corresponding statistics, obtained with R’s cocor package (Diedenhofen, 2019; using Pearson and Filon's z, 1898) for overlapping correlations, in the supplemental information.

R1. The authors do not explicitly show that the two kinds of neural representations are dissociable or that neural representations are sufficiently stable within conditions to be used in an RSA. One can imagine that representational drift across trials could lead to high variance of neural representations within a single condition, leading to low similarity scores within that condition and therefore deflated second–order similarity scores. To show that the neural representations of self threat and other's distress are dissociable, the authors would need to first determine the distributions of these two kinds of representations in conditions where subjects are faced only with a threat to themselves or only with the distress of another. This could be done, for example, by continuing to image while subjects rated self threat and other's distress and extracting the distribution of voxel patterns that are correlated with these ratings; then, methods from signal detection theory could be used to determine if the two distributions are separable.

We fully agree with the reviewer that it would be have been ideal to directly measure the neural representation of other’s distress and self threat by scanning participants while they provided ratings or distress and threat. The reason we decided not to do this is that it would have increased scanning time to 2 hours or more (the ratings were self-paced). Fatigue can compromise data quality, and we did not want to prolong the scanning time beyond what is reasonable to ask from participants. We therefore decided to obtain only behavioural representations of other’s distress and self threat outside of the scanner. We would like to point out that the use of behavioural models in RSA analysis has been a successful and frequently used approach in previous studies (e.g., Parkinson et al., 2014).

R1. The authors assume that when the threat and conspecific in need are presented simultaneously the neural representations in these contexts will be a linear combination of the separate representations for self threat and other's distress. Evidence from other domains suggests that may be unlikely; for example, in olfaction, sensory representations of mixtures of two odors are rarely a linear combination of the individual sensory representations of each odor presented separately.

We would like to clarify that we do not assume a linear combination of representations of threat and other’s distress. Indeed, no assumptions were made regarding how these representations are combined when both cues are present. Instead, our focus was to find the association between those representations and helping behaviour. We realize, based on the reviewer’s comment, that our main goal was not sufficiently clear in the manuscript, so we have now clarified it in the following way:

P.15: “To dissociate the role of representations of threat and of other’s distress on helping behaviour, after the scan we asked participants to rate…”.

P.17: “To decide whether to help another person in a dangerous situation, one must consider not only their distress, but also the threat in the environment. Here, we determined how the representations of another’s distress and of threat guide behaviour.”

R1. These conceptual issues with the RSA hinder its interpretation here and make it difficult to accept the authors' interpretation that "neural representations of threat promoted helping'.

We agree with the reviewer that some of these conceptual issues were not made sufficiently clear in the initial version, and are thankful to be given the opportunity to address them. We hope that with the current alterations and clarifications, the RSA analysis and its interpretation will be clearer to readers.

– Data Pooling/Muddling

R1. In the section "Greater engagement of reactive fear circuits led to helping", the authors pool imaging data from trials on which a "no help" decision was made with imaging data from safe trials. A decision under threat to help a conspecific and an arbitrary decision with no consequences for either the subject or conspecific should involve different neural mechanisms, so there is no clear justification for pooling the data from these two conditions. Pooling data from these two conditions makes it impossible to determine whether the results of the ANOVA provide sufficient evidence for their conclusion that "greater engagement of reactive fear circuits led to helping'.

R2. As mentioned in line 227, because of a reduced number of "no help" decision trials in the conditions where threat is present, the authors used trials from the safe condition in conjunction to form the regressor. This might reflect an "inflation" in "help" decisions, possibly due to the nature of the experimental environment and task design [See comments regarding 'instructions']. This could influence the interpretation of the behavioral results – for example, there was no significant difference between the "help" ration in distal and imminent threat conditions, and that could be due to the saturated "helping" responses (line 798, Figure 2A). This is also potentially problematic for the imaging analysis, since there has been research showing that safety signals are implicated in the vmPFC regions, which overlap with the key ROIs listed in this research. The "no help" trials under safety would not be homogenous to the "no help" trials under threat.

We appreciate the point made by both reviewers that there are limitations in combining “not help” and safe decisions in the comparison against help decisions. In fact, this was not our preferred approach, and it was made to overcome the reduced number of “not help” trials in comparison to help trials.

To address the reviewers’ concerns, we now included in the manuscript a parametric modulation analysis. Here, we used a GLM that included a parametric modulator for decisions made in shock trials (0 if no help, and 1 if helped). This analysis has the advantage of modelling all threat imminence and level conditions in the GLM (including safe trials), but only assigning parametric modulators to conditions in which a subsequent helping decision was made (i.e., distal 1 shock, distal 2 shocks, imminent 1 shock and imminent 2 shocks). The disadvantage of this approach is that only participants that at least 1 trial in which they did not help were included. The results of the parametric modulator analysis were consistent with our original findings. We have now added the following paragraph to the manuscript:

P. 12: “One limitation of this analysis is that it conflated responses made during safe trials and no help responses. It could be argued that these represent fundamentally different types of decisions. To overcome this limitation, we also performed a parametric modulator analysis, in which we used a GLM that included a parametric modulator for decisions made in shock trials (0 if no help, and 1 if helped). This analysis allowed us to model all threat imminence and level conditions, but only assign parametric modulators to those in which a subsequent help or no help decision was made (i.e., distal 1 shock, distal 2 shocks, imminent 1 shock and imminent 2 shocks). The disadvantage of this approach is that only participants with at least 1 “no help trial” trial per condition were included (N=28). Results of the parametric modulator analysis were consistent with those of the Anova (Table 3), in that we only found significant modulation of brain activation by subsequent decision during distal threats. Activation of bilateral insula was increased before helping decisions (Distal 2 shocks), and activation of the vmPFC was increased before not helping decisions (Distal 1 and 2 shocks).”

R2. Both in the Results and Discussion sections (line 300 – 314), the authors mentioned that the average activation patterns for imminent and distal threats were contrary to previous research of similar nature. One possible explanation the paper might have overlooked is that the analysis would be done without the independence of "help/no help" decisions. In the previous research the paper cited, there was only one possible choice when under threat. However, in the current study, "help" resembles an approach choice, while "no help" an avoidance choice (and possibly safety signal). combining them would confound the results.

This is an excellent point. We have added this possibility in our Discussion as follows:

P. 15: “These results are opposite to those from prior studies that manipulated the imminence of self-directed threats (28, 30, 31, 33). It is possible, however, that the disparity between our and previous findings is due to methodological reasons and does not reflect true differences in the processing of self- versus other-directed threats. For instance, in previous work only avoidance responses were made, whereas in our paradigm both help and no help decisions were possible (perhaps more analogous to an approach and avoidance option). Importantly, …”.

R1. The authors should re–run the analysis without pooling the safe and no–help conditions. If the authors are worried about a lack of data on ``no help' decisions, they could take a Bayesian approach to the analysis since Bayesian models are non–asymptotic and therefore do not make the same demands on sample size as frequentist approaches. Summary statistics for the distribution of no–help decisions should be included.

R2. The authors could consider separating the "help" and "no help" trials in a more stringent manner, or create some control scenarios where only one action is possible;

We have addressed this important point made by both reviewers in our additional parametric modulation analysis, which we described above. We have also included the response distributions in the Supplemental information. We believe this extra analysis addresses the reviewers’ concerns, but are open to performing additional Bayesian analyses if the reviewers and editors deem it necessary. It should be noted, however, that the Bayesian estimation of betas associated with not-helping decisions will still rely on a smaller number of trials than helping decisions.

R2. The study would benefit from some adjustment to the paradigm itself – to reduce the "inflation" in helping, and increase the effectiveness of different levels of threat;

We agree with the reviewer that our paradigm could be adjusted, and in future research, we will certainly use the reviewers’ feedback to improve the paradigm. Yet, we are confident in the value of the added analyses suggested by the reviewers in further strengthening our original conclusions.
